# Urban meadows as an alternative to short mown grassland: effects of composition and height on biodiversity

**DOI:** 10.1002/eap.1946

**Published:** 2019-07-22

**Authors:** Briony A. Norton, Gary D. Bending, Rachel Clark, Ron Corstanje, Nigel Dunnett, Karl L. Evans, Darren R. Grafius, Emily Gravestock, Samuel M. Grice, Jim A. Harris, Sally Hilton, Helen Hoyle, Edward Lim, Theresa G. Mercer, Mark Pawlett, Oliver L. Pescott, J. Paul Richards, Georgina E. Southon, Philip H. Warren

**Affiliations:** ^1^ Department of Animal and Plant Sciences University of Sheffield Sheffield S10 2TN United Kingdom; ^2^ College of Life and Natural Sciences University of Derby Derby DE22 1GB United Kingdom; ^3^ School of Life Sciences University of Warwick Coventry CV4 7AL United Kingdom; ^4^ Centre for Environmental and Agricultural Informatics Cranfield University Cranfield MK43 0AL United Kingdom; ^5^ Department of Landscape University of Sheffield Sheffield S10 2TN United Kingdom; ^6^ Cranfield Soil and Agrifood Institute Cranfield University Cranfield MK43 0AL United Kingdom; ^7^ Department of Architecture and Built Environment UWE Bristol Bristol BS16 1QY United Kingdom; ^8^ School of Geography University of Lincoln Lincoln LN6 7TS United Kingdom; ^9^ Centre for Ecology and Hydrology Wallingford OX10 8BB United Kingdom

**Keywords:** beetles, carbon, conservation planning, green infrastructure, insects, microbial diversity, nitrogen, overwintering, plant richness, urban ecology, urban parks

## Abstract

There are increasing calls to provide greenspace in urban areas, yet the ecological quality, as well as quantity, of greenspace is important. Short mown grassland designed for recreational use is the dominant form of urban greenspace in temperate regions but requires considerable maintenance and typically provides limited habitat value for most taxa. Alternatives are increasingly proposed, but the biodiversity potential of these is not well understood. In a replicated experiment across six public urban greenspaces, we used nine different perennial meadow plantings to quantify the relative roles of floristic diversity and height of sown meadows on the richness and composition of three taxonomic groups: plants, invertebrates, and soil microbes. We found that all meadow treatments were colonized by plant species not sown in the plots, suggesting that establishing sown meadows does not preclude further locally determined grassland development if management is appropriate. Colonizing species were rarer in taller and more diverse plots, indicating competition may limit invasion rates. Urban meadow treatments contained invertebrate and microbial communities that differed from mown grassland. Invertebrate taxa responded to changes in both height and richness of meadow vegetation, but most orders were more abundant where vegetation height was longer than mown grassland. Order richness also increased in longer vegetation and Coleoptera family richness increased with plant diversity in summer. Microbial community composition seems sensitive to plant species composition at the soil surface (0–10 cm), but in deeper soils (11–20 cm) community variation was most responsive to plant height, with bacteria and fungi responding differently. In addition to improving local residents’ site satisfaction, native perennial meadow plantings can produce biologically diverse grasslands that support richer and more abundant invertebrate communities, and restructured plant, invertebrate, and soil microbial communities compared with short mown grassland. Our results suggest that diversification of urban greenspace by planting urban meadows in place of some mown amenity grassland is likely to generate substantial biodiversity benefits, with a mosaic of meadow types likely to maximize such benefits.

## Introduction

Urban greenspace has the potential to support considerable biodiversity (Aronson et al. 2014, Beninde et al. 2015) with potential benefits for human well‐being (Fuller et al. 2007, Dallimer et al. 2012, Pett et al. 2016), ecosystem service provision (Tratalos et al. 2007, Radford and James 2013, Schwarz et al. 2017), and local and global conservation (Ives et al. 2016). With the growth of urban land cover globally (Seto et al. 2012), the role of cities in contributing to conservation and ecosystem service provision is increasing. The potential of urban areas to deliver these benefits is, however, being eroded by loss of greenspace to redevelopment and densification (Haaland and van den Bosch 2015) and by typical approaches to urban greenspace management (Aronson et al. 2017). Particularly common is the maintenance of greenspace as short mown grass in the form of lawns or amenity grassland (Müller et al. 2013). Amenity grassland is frequently mown, short sward vegetation that is managed for human recreational use, examples include lawns in public parks and sports grounds. Short‐mown grassland habitats dominate temperate cities, in both public and private urban greenspaces, for example they cover 22.5% of the land area of Swedish cities, almost double the cover 50 yr ago (Hedblom et al. 2017), and similar amounts in the UK (25%; Evans et al. 2009) and United States (23%; Robbins and Birkenholtz 2003), which equates to 1.9% of the total land area of the continental United States (Milesi et al. 2005).

Short‐mown urban grasslands are popular due to their assumed aesthetic value, well‐established and widely accepted management protocols, provision of recreational space and associated social norms (Harris et al. 2013, Ignatieva et al. 2015, Hoyle et al. 2017). However, they require intensive management, with UK local authorities typically mowing every 2–3 weeks during the growing season (March–September; Garbuzov et al. 2015) and the total number of annual cuts is increasing with extended growing seasons under climate change (Sparks et al. 2007). Many lawns and parks receive frequent inputs of fertilizer, herbicide, and, depending on local climate, irrigation (Alumai et al. 2009, Bertoncini et al. 2012, Bijoor et al. 2014). This is financially and environmentally costly (Smetana and Crittenden 2014), and at odds with reduced funding for managing public spaces in many developed regions (Walls 2009, Heritage Lottery Fund 2014).

Cumulatively across urban areas, parks can harbor significant numbers of plant species (Thompson et al. 2004, Stewart et al. 2009, Bertoncini et al. 2012), and, per unit area, lawns support species richness similar to those of seminatural grasslands, although composition is often dominated by a small number of grass species (Thompson et al. 2004, Bertoncini et al. 2012, Wheeler et al. 2017). However, the limited vegetation structure provided by short grass swards leads to reduced diversity of many invertebrate taxa relative to more structurally complex grasslands (Morris 2000, Jerrentrup et al. 2014). This results from direct effects of reduced habitat availability and complexity and other effects such as microclimate alteration (Gardiner and Hassall 2009), trampling by humans (Duffey 1975), and mowing limiting forb flowering and seed set (Garbuzov et al. 2015) and causing direct mortality (Humbert et al. 2010). As a result, there is growing interest around the world in finding more structurally and botanically diverse alternatives to mown amenity grassland (Bormann et al. 2001, Klaus 2013, Blackmore and Goulson 2014, Hwang et al. 2017, Jiang and Yuan 2017).

Introducing areas of “meadow” vegetation, broadly defined as infrequently mown grassland, usually with flowering forbs, to replace park grass is thought to ameliorate some of these effects. Meadow‐like areas can be established by reducing mowing frequency, allowing the existing plant community to increase in height and flower cover (Garbuzov et al. 2015, Wastian et al. 2016, Lerman et al. 2018). However, the outcome of this approach is dependent on the diversity of the existing flora, and any subsequent natural colonization. An alternative method for establishing meadows is deliberate seeding or planting of designed mixes of plant species. This latter approach to meadow creation predominantly uses annual plant species to enhance aesthetic value (Dunnett and Hitchmough 2007, Dunnett 2011), but may be complemented with perennial species to reduce the need for re‐sowing (Hoyle 2016). Urban meadow areas are widely advocated by conservation organizations (RSPB 2013, The Wildlife Trusts 2018). While the potential benefits to people and wildlife are widely articulated, and there are some studies of human responses (Jiang and Yuan 2017, Southon et al. 2017), there is little work quantifying the ecological effects of different types of urban meadows in public greenspaces (Klaus 2013). This contrasts with the more extensive examination of the ecological effects of increasing wildflower coverage in agricultural systems (Knop et al. 2006, Haaland et al. 2011, Buri et al. 2016). Urban meadows, however, warrant separate attention as conditions, and constraints, differ substantially from agricultural systems. Notably, soil conditions in urban and agricultural areas differ due to numerous factors including the absence of livestock or specialist management for crops, pollutant concentrations and different exposure to other management activities such as regular plowing (Pouyat et al. 1995, Setälä et al. 2016). There are also substantial differences in colonization potential as urban grasslands are often poorly connected (Hejkal et al. 2017), fragmented by urban land covers or other vegetation (Williams et al. 2009). Furthermore, the need to develop plant mixes that are acceptable to the public in areas close to housing presents particular challenges in introducing taller and “messier” vegetation (Hoyle et al. 2017).

Here, we use a replicated set of nine different perennial meadow treatments, sown in six public urban greenspaces in southern England, to quantify the relative roles of floristic diversity and height on the diversity and composition of plant, invertebrate and soil microbial communities. These results form part of a wider assessment of these meadow plantings, which include assessments of the responses of local residents (Southon et al. 2017, 2018) and greenspace managers (Hoyle et al. 2017).

## Methods

### Meadow establishment and experimental design

Meadow plots were established in areas of urban mown amenity grassland at sites adjacent to residential housing, on clay‐loam soils in five areas in Bedford (Chiltern Avenue, Jubilee Park, Goldington Green, Brickhill Heights) and Luton (Bramingham Road), in Southern England (Appendix S1: Fig. S1, Table S1). Meadows were also established on clay soils adjacent to campus residential housing at Cranfield University, situated in the countryside but with urban development features (high‐ and low‐rise buildings and housing, roads, airport; Appendix S1: Fig. S1). Meadows were hand sown in early May 2013 in plots rotovated to a depth of 100–150 mm (rotovating breaks the ground up and achieves a fine tilth for sowing, similar to tilling) after being treated with glyphosate herbicide. Some hand weeding was done on all plots in July 2013, targeting four species that became sources of complaints from local residents (all sites: *Chenopodium album*,* Sonchus oleraceus*, and *Helminthotheca echiodes*; Jubilee Park only: *Potentilla reptans*). Weeding was done across all sites, and although weeding effort was not quantified, it was not systematically related to treatments. In addition, to ensure successful establishment, all low diversity (grass) plots (except two; the tall plots at Goldington Green and Bramingham, which established adequately) were sprayed with herbicide and reseeded at higher density in autumn 2013, and bare patches in the medium and high diversity plots were over‐sown at the original density (Appendix S1: Table S2). One site (Jubilee Park) was rotovated and resown in April 2014 due to poor establishment. Plots were sampled in their second growing season (Jubilee Park, 2015; all other sites, 2014). Due to their smaller size, the Cranfield plots were only used when assessing soil properties.

**Figure 1:**
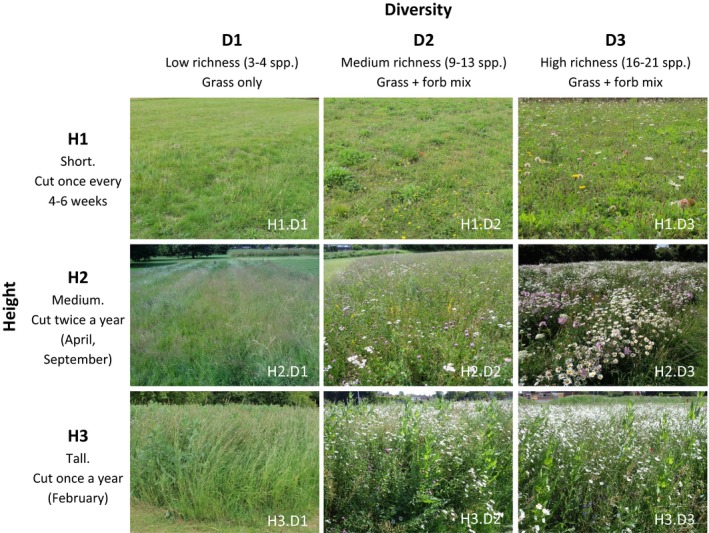
The nine experimental treatments shown across two axes of variation; height (H1, H2, H3) and diversity (D1, D2, D3), with example photographs taken in early summer of the second year after establishment. Diversity treatments differed in total species richness and relative proportion of forb and grass. Height treatments differed in mowing regimes as well as plant selection. Details of the nine seed mixes are in Appendix S1: Table S2. At each site, an area of the original mown amenity grassland equal in area to the treatment plots but without special management (the *unmanipulated control* plot) was also surveyed.

Nine meadow treatments spanned two axes of variation: plant species richness (low, medium, and high) and height (short, medium, and tall; Fig. 1). All sown species were perennials and native to southern England. Seed mixes for each treatment were randomly allocated to standardized rectangular plots, with at least 5‐m gaps of original short mown grass between plots. The arrangement of the plots in relation to each other varied between sites depending on site shape and existing infrastructure. Plots were 250 m^2^ (12.5 × 20 m), except at Cranfield where, due to space constraints, plots were 50 m^2^ (5 × 10 m). At all sites, an area of the original short mown grass equal in area to the treatment plots was identified and surveyed, but was subject to no preparatory cultivation and continued to be managed identically to the surrounding mown amenity grassland (referred to as the *unmanipulated control*).

Plant species richness was manipulated by sowing seed mixes (Appendix S1: Table S2) varying in total species richness and ratio of grass to forbs (broad‐leaved herbaceous plants; Fig. 1; Appendix S1: Table S2). The low plant species richness seed mixes contained only grasses and the short plots containing this mix thus simulated newly sown, mown, amenity grassland. Species composition of plots was chosen to achieve the target heights and flower cover for the different treatments under the proposed mowing regimes (see below paragraph), meaning that the composition had to vary somewhat between treatments (i.e., maintenance of high floral diversity in a tall plot requires different species from a short, regularly mown, high diversity plot).

Vegetation height was determined by choice of plant species and different cutting regimes: short plots were cut to a target height of 0.05 m every 4–6 weeks dependent on staffing and weather (Hoyle et al. 2017), medium height plots were cut twice a year (April and September), and tall plots were cut once a year (February). Medium plots had a target height of approximately 0.50 m during the growing season, and tall plots reached an average maximum height of 1.50 m. At most sites, all nine treatments were established (Appendix S1: Table S1). The exceptions were Goldington Green where, due to space constraints, only the low and high richness treatments were implemented, and Brickhill Heights, where four plots were discontinued due to feedback from residents (Hoyle et al. 2017), leaving two short and all tall treatments (Appendix S1: Table S1).

### Botanical surveys

Botanical surveys were conducted in July, in the second year after establishment (Jubilee Park, 2015; all other sites, 2014). Surveys were undertaken in five replicate 1‐m^2^ quadrats in all plots, arranged in a quincunx and at least 2 m from the plot edge. The percentage cover of each species was recorded on the Domin scale (Rodwell 2006) and an average calculated for each plot using the midpoints of the Domin categories. In each plot, species recorded were separated into sown and non‐sown, the latter classified as any species not in the seed mix for that plot (although it may occur in one of the other treatment seed mixes).

### Invertebrates

Aboveground invertebrates were sampled in all plots in summer (June 2014, July 2015) and early autumn (September 2014, 2015) using sweep‐nets and vacuum sampling. Overwintering invertebrates were sampled at four sites in February 2015, prior to tall plots being cut but after the medium plots were cut (September). Winter sampling involved time standardized searches that comprised a sequence of beating, cutting, collecting and sieving vegetation. A pooter/aspirator was used to extract invertebrates from the ground surface or sieved samples, by orally sucking them into collecting tubes. For all samples, we quantified the number of individuals and, for summer and autumn samples, the biomass in each invertebrate order (Appendix S1: Table S3). We use both abundance and biomass as these are not necessarily strongly correlated, for example, if high abundances are driven by large numbers of small‐bodied individuals. Coleoptera from both summer and autumn samples were identified to family at the three sites with a full set of treatments (Chiltern Avenue, Bramingham Road, Jubilee Park). Summer, autumn and winter samples were analysed separately as responses of some taxa to the meadow treatments may vary seasonally. Further details of the sampling methods are provided in the Appendix S1: Section S1.

### Soils

#### Soil sampling

All soil sampling and measurements were undertaken at Chiltern Avenue, Bramingham Road, and Cranfield, providing examples of all treatments, across the broadest range of soil types at sites with the full suite of treatments. Soils were sampled (33 mm internal diameter, gouge‐style auger) in February 2015 in each plot, including the unmanipulated control. Soil sampling locations were ascertained by splitting each plot into three subplots of equal size. Within each subplot, three samples were taken from randomly generated coordinates. These were then bulked at each of two depths (0–10 cm and 11–20 cm), providing three bulked cores for each plot and depth.

#### Soil total nitrogen and total carbon

The soil samples were prepared for analysis by homogenizing and sieving (2 mm) each sample. Total carbon (BS 7755‐3.8:1995) and total nitrogen (BS EN 13654‐2:2001) were assessed using the Elementar Vario III EL analyzer (Elementar, Langenselbold, Germany).

#### Soil biological community

Microbial biomass C was determined using the fumigation‐extraction procedure (Jenkinson and Powlson 1976) using a *K*_EC_ (extraction efficiency coefficient) of 0.45 (Vance et al. 1987). Microbial community phenotypic characteristics were determined by analyzing cellular phospholipids (based on Frostegård et al. 1993) and the relative abundance of indicator fatty acids for arbuscular mycorrhizal fungi and bacteria. Total DNA was extracted from 250 mg of homogenized soil and sequenced on the Illumina MiSeq (Illumina, inc., San Diego, California, USA) platform for fungal (ITS3 and ITS4) and bacterial (16S—515f and 806r) primers. Sequences were clustered to operational taxonomic units (OTUs) with a 97% minimum identity threshold (after excluding sequences that only occurred once). Taxonomy was assigned using quantitative insights into microbial ecology (QIIME 1.8; Caporaso et al. 2010) and the Greengenes reference database for 16S (McDonald et al. 2011), or the UNITE database for ITS (Kõljalg et al. 2013). Full details of the extraction and sequencing methods are provided in the Appendix S1: Section S2.

### Data analysis

All analyses were performed using R (version 3.5.0; R Core Team 2018) unless otherwise stated.

#### Plants and invertebrates: comparison of unmanipulated controls and short, low diversity treatments

We compared the response of plants and invertebrates to the short, low diversity (H1.D1) plots (that simulated newly sown mown amenity grassland) and the unmanipulated control plots with paired *t* tests using the PairedData package (Champely 2018). We compared the richness and cover of plants, the richness of invertebrate orders (all seasons) and Coleoptera families (summer and autumn), and total invertebrate abundance (all seasons).

#### Plants and invertebrates: effects of vegetation height and diversity treatments

Linear mixed effects models were constructed using the package lme4 (Bates et al. 2015) with height (three levels) and diversity (three levels) treatments included as fixed effects and site as a random intercept, using maximum likelihood parameter estimation. No interaction term was included as there was no within‐site replication. These analyses exclude the unmanipulated control plots. Response variables were transformed where necessary (Table 1). Significance of the main fixed effects was assessed with an ANOVA of the model output using the package lmerTest (Kuznetsova et al. 2017), with degrees of freedom based on Satterthwaite's approximation. Within‐treatment pairwise comparisons were determined with post‐hoc tests using least‐squares means in package lsmeans (Lenth 2016).

**Table 1:** Results of linear mixed models for plant community variables

				Contrasts		
Fixed effects variable	df	*F*	*P*	Low–medium	Low–high	Medium–high
All plants richness, square‐root transformed						
Diversity	2,34.0	5.80	**0.007**	**M > L**,** 0.044**	**H > L**,** 0.009**	0.942
Height	2,33.9	3.84	**0.031**	0.507	0.218	**M > H, 0.026**
Not‐sown plants richness, untransformed						
Diversity	2,34.0	1.70	0.197	0.991	0.263	0.283
Height	2,33.9	7.07	**0.003**	0.649	**L > H**,** 0.022**	**M > H**,** 0.003**
Not sown plants cover, 1/4‐power transformed						
Diversity	2,34.1	3.45	**0.043**	0.882	**L > H**,** 0.043**	0.189
Height	2,33.9	2.38	0.108	0.224	0.914	0.107

*Notes*

**Figure 2:**
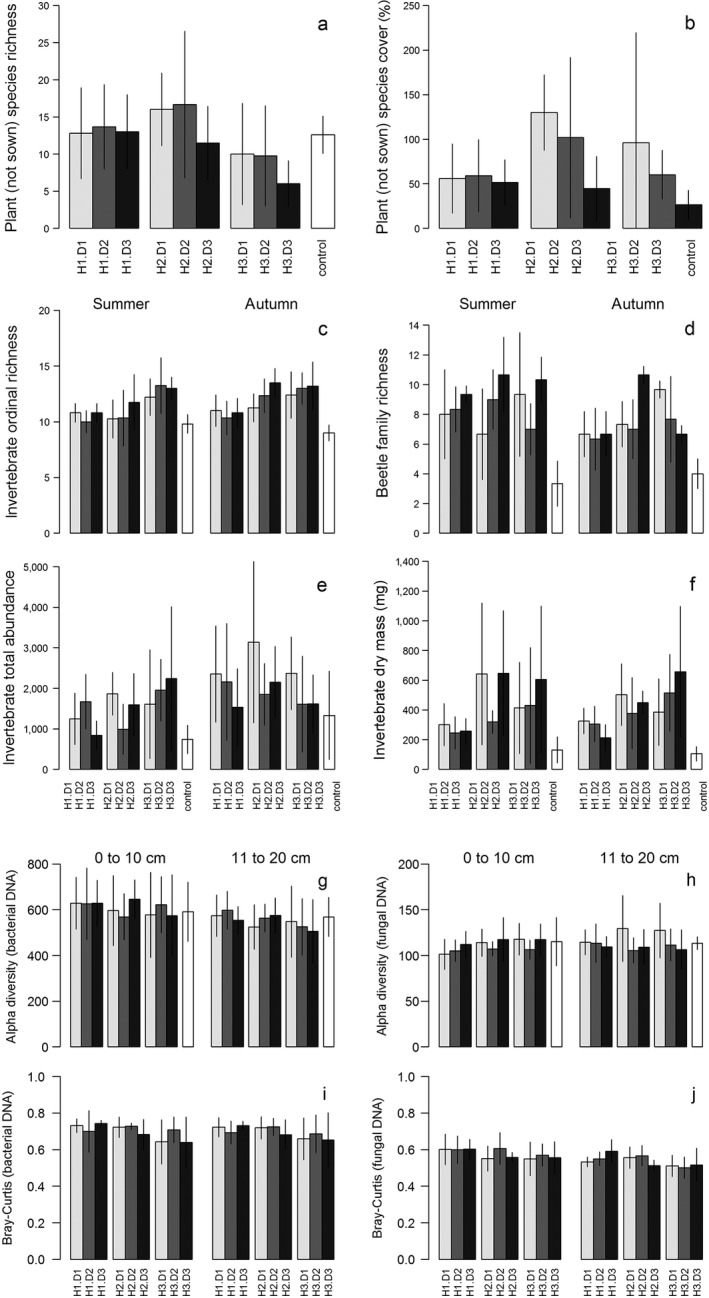
Plant and invertebrate community properties by treatment and by season for invertebrates and depth for the soil community. Treatment combinations along the *x*‐axis correspond to Fig. 1. Bars are organized from short (left) to tall (right) treatments, with small gaps between the height groups, and diversity treatment is indicated by gray shading; light gray, low diversity; medium gray, medium diversity; black, high diversity. White bars represent the unmanipulated control. Bars are the mean per treatment combination with standard deviation bars. Plants are represented by (a) non‐sown plant richness and (b) percent cover. The invertebrate community is represented by (c) order‐level richness, (d) Coleoptera family richness, (e) total community abundance, and (f) estimated total community biomass. The soil taxonomic community is represented by alpha diversity of (g) bacterial and (h) fungal operational taxonomic units (OTUs) and the difference of the (i) bacterial and (j) fungal DNA communities from the composition of the unmanipulated control. Error bars show ± SD.

Significant effects (*P* < 0.05) are indicated in boldface type. Contrasts are the results of least‐squares means (see subsection *Plants and invertebrates: effects of vegetation height and diversity treatments* for details). Where there was a significant difference between treatments, the direction of the effect is indicated. Refer to Fig. 2 for the differences between treatment means.

Models were constructed for three plant responses: richness of all plant species, richness of non‐sown species, and cover of non‐sown species. The response variables for the invertebrate models for all seasons (summer, autumn, winter) were richness (order level) and total abundance. For summer and autumn, invertebrate sample models were also run for Coleoptera richness (family level), total biomass (dry mass), and abundance of each order with more than 1,000 individuals (Appendix S1: Table S4). Data from summer, autumn, and winter were analysed separately.

#### Plants and invertebrates: ordinations

To assess the effect of height and diversity treatments on the composition of the biological communities, data from the nine experimental treatments were ordinated using nonmetric multidimensional scaling using metaMDS in the vegan package (Oksanen et al. 2017). Ordinations were run for the non‐sown plant community, the order‐level invertebrate community (summer and autumn) and family‐level Coleoptera community (summer and autumn). Invertebrate data from summer and autumn were assessed separately. Data were square‐root transformed and submitted to Wisconsin double standardization (Oksanen et al. 2017). Pairwise dissimilarities were calculated using the Bray‐Curtis index. A maximum of 50 random starting configurations were used to find a stable solution. If stress was greater than 0.2 with two axes, a third axis was added. The function adonis in vegan was used to compare the location of the centroid of each tested group statistically, applying nonparametric permutational ANOVA using dissimilarity matrices (Oksanen et al. 2017). Data met the adonis test assumptions of homogeneity of variance (tested using betadisper in vegan) with the exception of the non‐sown plants and Coleoptera community in summer (Appendix S1: Table S5). Models were constructed including height and diversity blocked by site, with Bray‐Curtis dissimilarities. As explanatory variables are entered sequentially, each model was run twice, reversing the order that height and diversity treatments were entered into the model, and results for each variable are reported for the model where the other variable has been taken into account.

#### Soils: effects of vegetation height and diversity treatments

Linear mixed effects models of total nitrogen and total carbon and four measures of the microbial community (see below paragraph) were constructed in JMP (Version 13.0; SAS Institute Inc., Cary, North Carolina, USA). Height treatments (three levels), diversity treatments (three levels) and an interaction term between height and diversity were included as fixed effects. Replicates within each plot were nested within site as a random effect, corresponding to the three cores obtained from each plot. This approach reflects differences in the sampling design used for soil microbes and the invertebrate and plant communities. Parameter estimation was undertaken with restricted maximum likelihood (REML). Each response variable was analysed separately for 0–10 cm and 11–20 cm depths.

The microbial community was characterized with phospholipid fatty acid analysis (PLFA) and DNA. The microbial community of each plot, including the unmanipulated control plots, as characterized by PLFA was ordinated using principal components analysis, in JMP (Version 13.0; SAS Institute) on the correlation matrix using a standard least squares estimator. The first and second principal components were extracted for further analysis. The community, as characterized by its DNA, was first separated into fungal (Kingdom Fungi) and bacterial (Domain Bacteria) components. Each community was analysed at the OTU level. Fisher's alpha diversity (Fisher et al. 1943) of the bacterial and fungal communities was calculated for each plot. The community in each treatment plot was characterized by its difference from the unmanipulated control plot at the same site using Bray‐Curtis dissimilarities (Bray and Curtis 1957). These six variables (PLFA principal components one and two; bacterial and fungal DNA alpha diversity; bacterial and fungal DNA Bray‐Curtis dissimilarity), measured at both depths, were used as response variables in linear models.

## Results

### Plants

A total of 106 plant species were detected across all the surveyed plots. Of these, only 33 were sown, and two were unique to unmanipulated control plots. Five sown species were not detected: *Anthriscus sylvestris*,* Hypericum perforatum*,* Primula veris*,* Ranunculus acris*, and *Tanacetum vulgare*. The three species with the greatest total cover were *Lolium perenne*,* Achillea millefolium*, and *Leucanthemum vulgare*. At the three sites with the full nine set of treatments, there were 35 (Chiltern Avenue), 47 (Jubilee Park), and 61 (Bramingham Road) non‐sown taxa detected. Averaging across all the sites, there were between four and eight non‐sown species per treatment plot. At the sites with the full nine set of treatments, most (between 79% and 84%) of the non‐sown taxa were not found in the unmanipulated control plot; similar proportions (74% and 78%) were observed at sites with fewer plots.

The unmanipulated control plot and short, low diversity, treatment (H1.D1) did not differ significantly in total richness (*t*_4_ = 8.85, *P* = 0.441) or cover (*t*_4_ = 0.96, *P* = 0.387). Overall, total plant richness follows the pattern established by the diversity treatments (Tables 1, 2). Richness of non‐sown plants varied significantly with height treatments, where tall plots had significantly lower non‐sown plant richness than medium or short plots (Fig. 2a; Table 1). Cover of the non‐sown species varied with diversity treatment, with the high diversity treatments having significantly lower cover of non‐sown species (Fig. 2b; Table 1).

**Table 2:** Summary of the response of plant and invertebrate richness, abundance, and composition and of individual invertebrate orders to the two axes of experimental meadow treatments: diversity (three levels) and height (three levels)

Response variable	Direction of effect	
	Diversity	Height
Plants		
All plants richness	↑, L < M/H	∩, M > H
All plants composition	yes, NA	yes, NA
Not‐sown plants richness		↓, H < L/M
Not‐sown plants cover	↓, L > H	
Not‐sown plants composition	yes†, NA	yes†, NA
Invertebrates, Summer		
Order richness		↑, H > L/M
Order composition		yes, NA
Coleoptera richness	↑, H > L/M	
Coleoptera composition		
Invertebrate abundance		↑, H > L
Invertebrate biomass		↑, L < M/H
Invertebrate orders, Summer		
Acari		
Araneae		↑, H > L
Coleoptera	↑, H > L	↑, L < M/H
Collembola		
Diptera	↑, H > L	↑, H > L/M
Hemiptera	↓, L > H	↑, L < M/H
Hymenoptera		↑, H > L
Thysanoptera		↑, H > L
Invertebrates, Autumn		
Order richness		↑, L < M/H
Order composition		yes, NA
Coleoptera richness		
Coleoptera composition		yes, NA
Invertebrate abundance	↓, L > H	
Invertebrate biomass		↑, L < M/H
Invertebrate orders, Autumn		
Acari		
Araneae		
Coleoptera		↑, L < M/H
Collembola		
Diptera		
Hemiptera	↓, L > M/H	∩, M > L/H
Hymenoptera		∩, M > L/H
Psocodea		↑, H > M > L
Thysanoptera	↓, L > M/H	↑, H > L
Invertebrates, Winter		
Order richness		↑, H > L
Invertebrate abundance		↑, H > L

*Notes*

Results summarize linear model outputs (full results in Tables 1 and 3) and adonis results (full results in Appendix S1: Table S5). Here, significant effects for compositional change are indicated by “yes” and the direction effects are not applicable (NA). Significant effects for diversity and abundance measures are designated with a symbol indicating the direction of effect and text with detail of which treatments were higher or lower. An up arrow indicates the response variable increases with increasing meadow height/diversity. A down arrow indicates the response variable decreases with increasing meadow height/diversity. A ∩ symbol indicates that the response variable was highest in the medium height/diversity treatments. Non‐significant responses are left blank and indicate no detectable response to the treatment.

Note that data did not meet the adonis test assumptions of homogeneity of variance (tested using betadisper).

**Figure 3:**
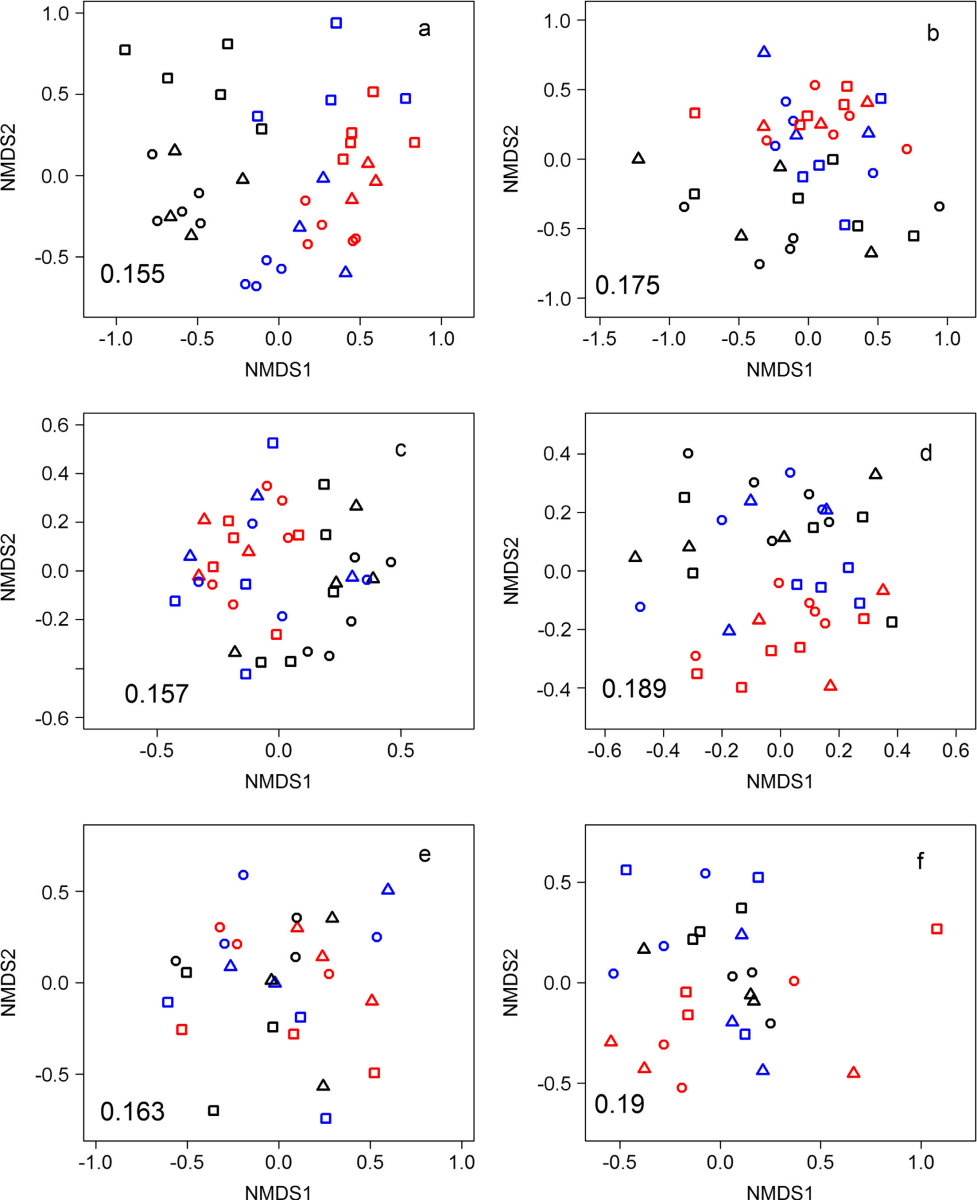
Nonmetric multidimensional scaling ordinations of (a) the whole plant community, (b) the non‐sown plant community, (c) the order‐level invertebrate community in summer and (d) in autumn, and (e) the Coleoptera family‐level community in summer and (f) in autumn. Points represent communities in each plot, coded by height (red, H1 [short]; blue, H2 [medium]; black, H3 [tall]) and diversity (square, D1 [low]; triangle, D2 [medium]; circle, D3 [high]). Stress values are in the bottom left of the plot space. Only the first two axes (NMDS1 and NMDS2) are shown, although all ordinations required three axes to reduce stress to <0.20, except for the order and Coleoptera communities in autumn.

Analysis with adonis showed that plant community composition, across all plant species, was significantly affected by height (*F*_9,37 _= 2.02, *P* = 0.001, *R* ^2^ = 0.32) and diversity treatments (*F*_9,37 _= 1.77, *P* = 0.001, *R* ^2^ = 0.28). Community composition of non‐sown plant species was significantly affected by diversity treatments (*F*_9,37_ = 1.24, *P* = 0.045, *R* ^2^ = 0.23) and height (*F*_9,37_ = 1.32, *P* = 0.018, *R* ^2^ = 0.24), although the result for non‐sown plants for height should be treated cautiously as there is heterogeneity in variance across the height treatments (*P* = 0.01; Fig. 3a, b; Appendix S1: Table S5).

### Invertebrates

In summer and autumn, over 138,000 invertebrates were collected from the treatment plots (excluding the unmanipulated control plots). The most abundant taxa were Collembola (45,151), Acari (24,502), Hemiptera (20,253), Diptera (13,662), and Coleoptera (11,048 of which 9,477 were adults). A total of 7,172 Coleoptera adults were sorted to family. Twenty‐three families were identified, of which the Nitidulidae (3,690), Latridiidae (781), and Staphylinidae (681) were most abundant. In winter, over 4,400 invertebrates were collected from a subset of plots (Appendix S1: Tables S1, S4). The most abundant taxa were Collembola (1,724), Diptera (705), and Gastropoda (455).

#### Community‐level richness and abundance

The invertebrate communities in the unmanipulated control plots and short, low diversity treatments (H1.D1) were not significantly different in summer (order richness: *t*_4_ = 1.58, *P* = 0.189; total abundance: *t*_4_ = 2.30, *P* = 0.083; Coleoptera family richness: *t*_2_ = 2.14, *P* = 0.166). In autumn, richness and abundance were higher in H1.D1 than the unmanipulated control plots (order richness: *t*_4_ = 3.65, *P* = 0.022; total abundance: *t*_4_ = 4.12, *P* = 0.014; Coleoptera family richness: *t*_2_ = 8.00, *P* = 0.015). In winter, abundance was not significantly different between H1.D1 and the unmanipulated control (*t*_2_ = 2.82, *P* = 0.106) but order richness was higher in H1.D1 plots (*t*_2_ = 4.91, *P* = 0.040).

Invertebrate responses varied between autumn and summer. Order‐level richness was affected by height treatment in both seasons (Fig. 2c; Tables 2, 3); tall plots had significantly higher richness than either short or medium height plots in summer, while in autumn, short plots had significantly lower richness than other height treatments. Coleoptera family richness responded only to diversity in summer, when the high diversity treatments had the highest richness (Fig. 2d). Invertebrate abundance (Fig. 2e) and biomass (Fig. 2f) both varied with height treatment in summer, as did total invertebrate biomass in autumn. The short plots drove differences in invertebrate biomass, having lower invertebrate biomass than either medium height or tall plots, while abundance in summer was significantly lower in the short than the tall plots. Invertebrate abundance was significantly higher in low than high diversity plots in autumn (Table 3). In winter, both order‐level richness and total abundance were significantly higher in the tall plots compared to the short plots (Table 3; Appendix S1: Fig. S2).

**Table 3:** Results of linear mixed models for invertebrate community variables in summer, autumn, and winter and for individual orders^a^ in summer and autumn

Fixed effects variable	df	*F*	*P*	Contrasts		
				Low‐medium	Low‐high	Medium‐high
Order richness						
Summer, ln(*x* + 1)‐transformed						
Diversity	2,41.5	0.97	0.388	1.000	0.433	0.508
Height	2,41.5	16.22	**<0.001**	0.891	**H > L**,** <0.0001**	**H > M**, <**0.001**
Autumn, untransformed						
Diversity	2,41.2	2.86	0.069	0.340	0.059	0.750
Height	2,41.2	17.41	**<0.001**	**M > L**, <**0.001**	**H > L**,** <0.0001**	0.921
Winter, ln(*x* + 1)‐transformed						
Diversity	2,25.0	0.88	0.426	0.476	0.836	0.795
Height	2,25.0	6.73	**0.005**	0.979	**H > L**,** 0.019**	0.068
Coleoptera, family richness						
Summer, untransformed						
Diversity	2,24.0	6.65	**0.005**	0.984	**H > L**,** 0.009**	**H > M**,** 0.014**
Height	2,24.0	0.14	0.874	0.938	0.867	0.984
Autumn, untransformed						
Diversity	2,27.0	0.88	0.426	0.536	0.990	0.457
Height	2,27.0	2.62	0.091	0.098	0.206	0.914
Invertebrate abundance						
Summer, ln(*x* + 1)‐transformed						
Diversity	2,41.4	0.11	0.892	0.992	0.931	0.897
Height	2,41.4	5.35	**0.009**	0.538	**H > L**,** 0.007**	0.128
Autumn, square‐root‐transformed						
Diversity	2,42.1	4.05	**0.025**	0.107	**L > H**,** 0.028**	0.927
Height	2,42.1	2.93	0.064	0.059	0.792	0.198
Winter, 1/4‐power‐transformed						
Diversity	2,25.0	0.85	0.440	0.510	0.719	0.917
Height	2,25.0	6.80	**0.004**	0.983	**H > L**,** 0.018**	0.065
Invertebrate biomass						
Summer, ln(*x* + 1)‐transformed						
Diversity	2,33.6	2.66	0.085	0.187	0.871	0.079
Height	2,33.6	6.82	**0.003**	**M > L**,** 0.006**	**H > L**,** 0.016**	0.822
Autumn, ln(*x* + 1)‐transformed						
Diversity	2,38.0	0.00	0.996	0.997	0.997	1.000
Height	2,38.0	5.34	**0.009**	**M > L**,** 0.040**	**H > L**,** 0.012**	0.941
Acari						
Summer, 1/4‐power‐transformed						
Diversity	2,33.4	0.03	0.972	0.983	0.998	0.971
Height	2,33.4	0.73	0.490	0.781	0.461	0.897
Autumn, square‐root‐transformed						
Diversity	2,33.6	0.08	0.924	0.967	0.920	0.994
Height	2,33.6	0.36	0.701	0.990	0.704	0.813
Araneae						
Summer, 1/4‐power‐transformed						
Diversity	2,33.5	0.73	0.492	0.940	0.470	0.753
Height	2,33.5	4.18	**0.024**	0.610	**H > L**,** 0.020**	0.213
Autumn, square‐root‐transformed						
Diversity	2,38.0	0.76	0.473	0.501	0.607	0.964
Height	2,38.0	2.09	0.138	0.236	0.165	0.995
Coleoptera						
Summer, ln(*x* + 1)‐transformed						
Diversity	2,33.0	3.62	**0.038**	0.784	**H > L**,** 0.034**	0.226
Height	2,32.9	9.28	**0.001**	**M > L**,** 0.013**	**H > L**,** 0.001**	0.678
Autumn, 1/4‐power‐transformed						
Diversity	2,33.2	1.78	0.184	0.648	0.159	0.709
Height	2,33.1	15.99	**<0.001**	**M > L**,** <0.0001**	**H > L**, <**0.001**	0.640
Collembola						
Summer, 1/3‐power‐transformed						
Diversity	2,33.9	1.21	0.311	0.977	0.318	0.517
Height	2,33.8	1.42	0.255	0.392	0.282	0.992
Autumn, ln(*x* + 1)‐transformed						
Diversity	2,34.3	2.22	0.124	0.362	0.118	0.893
Height	2,34.2	2.22	0.124	0.870	0.119	0.343
Diptera						
Summer, ln(*x* + 1)‐transformed						
Diversity	2,38.0	4.37	**0.020**	1.000	**H > L**,** 0.032**	0.054
Height	2,38.0	4.85	**0.013**	0.994	**H > L**,** 0.023**	**H > M**,** 0.039**
Autumn, ln(*x* + 1)‐transformed						
Diversity	2,34.1	1.55	0.227	0.271	0.347	0.950
Height	2,34.1	1.19	0.316	0.879	0.547	0.309
Hemiptera						
Summer, 1/5‐power‐transformed						
Diversity	2,38.0	2.83	**0.071**	0.622	**L > H**,** 0.058**	0.442
Height	2,38.0	16.92	**<0.001**	**M > L**,** 0.001**	**H > L**,** <0.0001**	0.351
Autumn, 1/5‐power‐transformed						
Diversity	2,33.9	7.41	**0.002**	**L > M**,** 0.026**	**L > H**,** 0.003**	0.867
Height	2,33.8	5.24	**0.010**	**M > L**,** 0.020**	1.000	**M > H**,** 0.018**
Hymenoptera						
Summer, ln(*x* + 1)‐transformed						
Diversity	2,33.8	2.65	0.086	0.930	0.087	0.264
Height	2,33.8	3.5	**0.041**	0.447	**H > L**,** 0.032**	0.435
Autumn, ln(*x* + 1)‐transformed						
Diversity	2,34.3	0.08	0.924	0.970	0.982	0.917
Height	2,34.1	10.46	**<0.001**	**M > L**,** 0.001**	0.965	**M > H**,** 0.001**
Psocodea						
Autumn, 1/5‐power‐transformed						
Diversity	2,38.0	0.74	0.483	0.451	0.869	0.732
Height	2,38.0	34.33	**<0.001**	**M > L**, <**0.001**	**H > L**,** <0.0001**	**H > M**,** 0.004**
Thysanoptera						
Summer, ln(*x* + 1)‐transformed						
Diversity	2,33.4	2.86	0.071	0.937	0.074	0.231
Height	2,33.4	13.52	**<0.001**	0.056	**H > L**,** <0.0001**	0.051
Autumn, 1/4‐power transformed						
Diversity	2,33.8	17.17	**<0.001**	**L > M**, <**0.001**	**L > H**,** <0.0001**	1.000
Height	2,33.8	5.58	**0.008**	0.330	**H > L**,** 0.006**	0.228

*Notes*

Significant effects (*P* < 0.05) are indicated in boldface type. Contrasts are the results of least‐squares means (see subsection *Plants and invertebrates: effects of vegetation height and diversity treatments* for details). Where there was a significant difference between treatments, the direction of the effect is indicated. For analyses of separate orders, only results for taxa with more than 1,000 individuals are presented (Appendix S1: Table S4). Refer to Fig. 2 for the differences between treatment means.

a Taxa with fewer than 1,000 individuals in total and not included here: Chilopoda, Diplopoda, Gastropoda, Dermaptera, Diplura, Ephemeroptera, Isopoda, Lepidoptera, Neuroptera, Odonata, Opiliones, Orthoptera, Psocodea (summer), Zygentoma.

#### Community composition

The composition of the invertebrate community at the order level was significantly affected by height treatment in both summer (*F*_9,37 _= 1.70, *P* = 0.026, *R* ^2^ = 0.25) and autumn (*F*_9,37_ = 1.76, *P* = 0.023, *R* ^2^ = 0.26) but not by diversity treatments in either season (Fig. 3c, d; Appendix S1: Table S5). Coleoptera community composition was only significantly affected by height treatment in autumn (*F*_6,26_ = 2.02, *P* = 0.007, *R* ^2^ = 0.33; Fig. 3e, f; Appendix S1: Table S5). There was no effect of plant diversity on community composition in summer or autumn, or of plant height on composition in summer, although the latter result should be treated cautiously as there is heterogeneity in variance across the height treatments (*P* = 0.01; Appendix S1: Table S5).

The effect of height and plant diversity on invertebrate abundance varied across taxonomic groups (Tables 2, 3). Differences in abundance were most pronounced between height treatments. Almost all groups had higher abundance in medium or tall plots than short plots, in at least one season (summer or autumn). The exceptions were the two taxa predominately found in soil, Acari and Collembola, which did not respond to vegetation height. Some taxa were more abundant in both medium and tall plots than short plots (both seasons: Coleoptera; summer: Hemiptera; autumn: Psocodea), some taxa were more abundant in medium plots than short plots (autumn: Hemiptera and Hymenoptera) and other taxa were more abundant only in tall plots compared to short plots (both seasons: Thysanoptera; summer: Araneae, Diptera, Hymenoptera). Significant effects of the diversity treatment on the abundance of individual orders occurred only for Coleoptera (positive; summer), Hemiptera (negative; summer and autumn), Thysanoptera (negative; autumn) and Diptera (positive; summer).

### Soils

#### Total nitrogen and total carbon

Total N varied with height treatments at both depths (*P* = 0.037 at 0–10 cm; *P* = 0.034 at 11–20 cm), where the medium plots had the highest total N (0–10 cm, $\overset{¯}{x}$ = 0.42, SE = 0.02; 11–20 cm, $\overset{¯}{x}$ = 0.25, SE = 0.01), followed by the short plots (0–10 cm, $\overset{¯}{x}$ = 0.4, SE = 0.01; 11–20 cm, $\overset{¯}{x}$ = 0.23, SE = 0.01) and tall plots had the lowest total N (0–10 cm, $\overset{¯}{x}$ = 0.34, SE = 0.01; 11–20 cm, $\overset{¯}{x}$ = 0.22, SE = 0.01; Appendix S1: Fig. S3). At both depths, total C was highest in high diversity plots (0–10 cm, $\overset{¯}{x}$ = 5.13, SE = 0.28, *P* = 0.024; 11–20 cm, $\overset{¯}{x}$ = 3.6, SE = 0.28, *P* = 0.01). The response of total C to the low and medium diversity plots varied with depth: at 0–10 cm, total C was higher in low diversity plots ($\overset{¯}{x}$ = 4.41, SE = 0.13, *P* = 0.005) compared to medium diversity plots ($\overset{¯}{x}$ = 4.48, SE = 0.13); at 11–20 cm, medium diversity plots had higher ($\overset{¯}{x}$ = 2.79, SE = 0.12, *P* = 0.004) total C than low diversity plots ($\overset{¯}{x}$ = 2.85, SE = 0.09; Appendix S1: Fig. S3).

#### Microbial community

**Figure 4:**
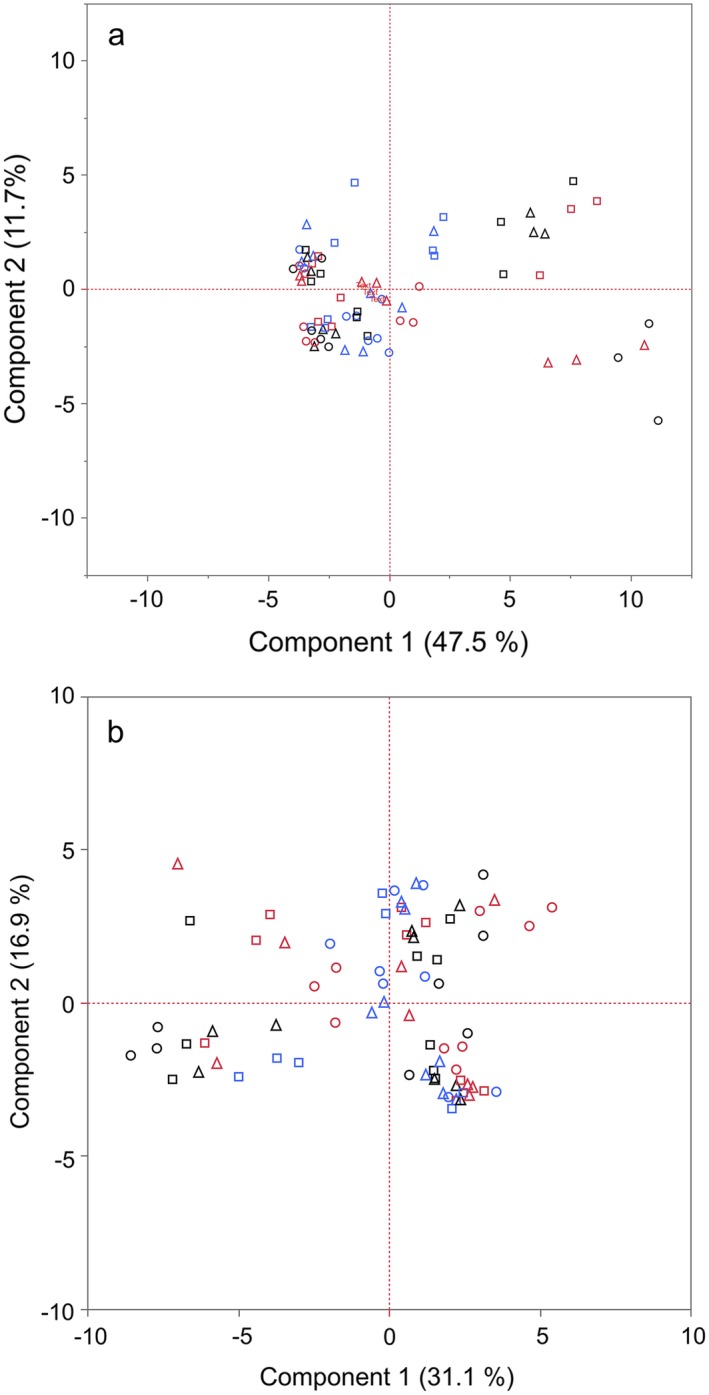
Biplots of the principal component scores of the phospholipid fatty acid profiles of the soil microbial community at (a) 0–10 cm depth and (b) 11–20 cm depth. The percentages on the axes refer to the variance described by the component represented on that axis. Points represent communities in each plot, coded by height (red, H1 [short]; blue, H2 [medium]; black, H3 [tall]) and diversity (square, D1 [low]; triangle, D2 [medium]; circle, D3 [high]).

The soil microbial community had different structure in shallow (0–10 cm) and deep (11–20 cm) layers. The first principal component representing the PLFA data was significantly affected by height treatment at both depths and there was a significant interaction with diversity treatment at both depths (Fig. 4, Tables 4, 5). The second principal component representing the PLFA data was significantly affected by diversity treatment at both depths and there was a significant interaction with height treatment at both depths (Fig. 4, Table 4).

**Table 4:** Results of linear mixed models for soil organism community variables at two depths (0–10 cm, 11–20 cm)

Random effects	Wald *P*	Fixed effects	df	*F*	*P*
0–10 cm depth					
PLFA principal component 1					
Plot	0.89	diversity	2,2	1.28	0.48
Site	0.32	height	2,2	6.94	**0.01**
		diversity × height	4,4	4.08	**0.01**
PLFA principal component 2					
Plot	**<0.0001**	diversity	2,2	21.33	**<0.0001**
Site	0.35	height	2,2	5.63	0.01
		diversity × height	4,4	17.55	**<0.0001**
Bacterial DNA alpha diversity					
Plot	0.31	diversity	2,2	0.65	0.53
Site	0.21	height	2,2	1.03	0.36
		diversity × height	4,4	0.84	0.50
Fungal DNA alpha diversity					
Plot	0.35	diversity	2,2	2.60	0.082
Site	0.58	height	2,2	3.34	**0.04**
		diversity × height	4,4	0.7	0.59
Bacterial DNA Bray‐Curtis					
Plot	0.965	diversity	2,2	0.55	0.582
Site	0.381	height	2,2	4.77	**0.012**
		diversity × height	4,4	1.32	0.277
Fungal DNA Bray‐Curtis					
Plot	**0.04**	diversity	2,2	3.41	**0.04**
Site	0.32	height	2,2	0.86	0.43
		diversity × height	4,4	3.18	**0.02**
11–20 cm depth					
PLFA principal component 1					
Plot	0.32	diversity	2,2	1.28	0.28
Site	0.27	height	2,2	6.94	**0.001**
		diversity × height	4,4	4.08	**0.004**
PLFA principal component 2					
Plot	0.37	diversity	2,2	5.76	**0.004**
Site	0.32	height	2,2	1.58	0.21
		diversity × height	4,4	2.93	**0.02**
Bacterial DNA alpha diversity					
Plot	0.32	diversity	2,2	0.78	0.46
Site	0.39	height	2,2	3.65	**0.03**
		diversity × height	4,4	0.95	0.44
Fungal DNA alpha diversity					
Plot	0.39	diversity	2,2	4.29	**0.02**
Site	0.45	height	2,2 = 0.13	0.13	0.87
		diversity × height	4,4	0.84	0.50
Bacterial DNA Bray‐Curtis					
Plot	0.58	diversity	2,2	4.14	0.77
Site	0.33	height	2,2	0.29	**0.02**
		diversity × height	4,4	1.67	0.17
Fungal DNA Bray‐Curtis					
Plot	0.04	diversity	2,2	0.21	0.81
Site	0.34	height	2,2	4.91	**0.01**
		diversity × height	4,4	2.01	0.10

*Notes*

Significant effects (*P* < 0.05) are indicated in boldface type. Where there was a significant difference between treatments, the direction of the effect is indicated. Refer to Fig. 2 for the differences between treatment means.

**Table 5:** Summary of the response of soil organism diversity and composition at two depths (0–10 cm, 11–20 cm) to the two axes of experimental meadow treatments: diversity and height

Measure	Effect		
	Diversity	Height	Interaction
Soils, 0–10 cm			
PLFA composition PC1		yes	yes
PLFA composition PC2	yes		yes
Bacterial DNA alpha diversity			
Fungal DNA alpha diversity		↑	
Bacterial DNA composition BC		yes	
Fungal DNA composition BC	yes		yes
Soils, 11–20 cm			
PLFA composition PC1		yes	yes
PLFA composition PC2	yes		yes
Bacterial DNA alpha diversity		↓	
Fungal DNA alpha diversity	↓		
Bacterial DNA composition BC		yes	
Fungal DNA composition BC		yes	

*Notes*

Significant effects for compositional change are indicated by “yes.” Significant effects for alpha diversity are designated with a symbol indicating the direction of effect; an up arrow indicates the response variable increases with increasing meadow height/diversity, a down arrow indicates the response variable decreases with increasing meadow height/diversity. Full results are in Table 4.

The bacterial community composition was characterized both as a function of alpha diversity and divergence of community composition from the unmanipulated control, measured as Bray‐Curtis dissimilarity. In terms of the alpha diversity, there was no significant effect on bacteria of either diversity or height treatment at either depth with the exception of a significant effect of height at the 11–20 cm depth (Fig. 2g, Tables 4, 5), which was driven by higher bacterial biodiversity for the short treatments. For fungal alpha diversity, there was a weak significant effect of height treatment at 0–10 cm, and taller plots were associated with higher fungal diversity. At 11–20 cm, there was a significant effect of diversity, and the low plant richness treatment was associated with higher fungal diversity (Fig. 2h, Table 4). For bacteria, Bray‐Curtis dissimilarity analysis showed that height had a significant effect on community composition at both depths (0–10 cm and 11–20 cm; Fig. 2i, j, Table 4). Fungal community composition was significantly affected by diversity at 0–10 cm depth, and height at 11–20 cm depth, and furthermore there was an interactive effect of height and floristic diversity on fungal community composition at 0–10 cm.

## Discussion

We replaced mown amenity grassland with a range of meadow‐type vegetation in six public greenspaces to assess the response of biological diversity to urban meadow habitat creation. We found that increasing the height and sown species diversity of the plant community generally altered the composition of soil microbial and aboveground invertebrate communities, increased invertebrate biomass, abundance, and richness and reduced incursion by non‐sown plant species. Increasing plant height was associated with lower richness of non‐sown plants and higher richness, biomass, and abundance of invertebrates, although responses of individual taxa varied. Increased height also changed composition of the soil bacterial community at 0–10 cm and 11–20 cm depth, and of soil fungal communities at 11–20 cm. Increasing plant diversity and forb to grass ratio was associated with lower cover of non‐sown plants, greater beetle richness in summer, greater abundance of some invertebrate orders and changed the composition of the soil fungal community at 0–10 cm depth. Overall, the meadows increased biodiversity but the varying responses of the different taxonomic groups to the treatments suggests that maintaining a diverse range of meadow types within a site or across a network of sites would be most beneficial for urban green space biodiversity.

### Plants

Non‐sown species contributed substantially to the plant communities of all treatment plots. The vast majority of non‐sown species (approximately 75%) were not found in the unmanipulated control plots at the same site. While this may in part be related to survey area (species found in single 250‐m^2^ control plots are unlikely to include all those present in this site), it does suggest that many of the non‐sown species have not colonized directly from a site's mown amenity grassland species pool. A large proportion (67%) of the non‐sown species had ruderal tendencies (Grime 1979) and within these, 41% had annual lifecycles (Appendix S1: Table S6; Grime 1979, Grime et al. 1995). Many of these produce large quantities of seed that disperse widely (Grime 1977), and they may have colonized by this route; however, ruderals may also have seeds that remain viable in seed banks for many decades (Thompson et al. 1997) and soil disturbance during plot cultivation may have stimulated their germination. Higher cover of ruderal species has also been observed early in the restoration of agricultural areas with meadow‐like vegetation (Pywell et al. 2011).

The number of colonizing species was lower in taller plots, and their cover was lowest in the plots with the highest diversity treatment. In our study system, ecological conditions at the time of meadow establishment were similar across all treatment plots, and there were consistently low levels of bare ground during the survey period (Appendix S1: Section S3, Figure S4). In addition, the potential pool of incoming species should be relatively consistent across plots within each site. This suggests that a combination of the diversity of competitors, and factors associated with vegetation structure (light availability being the most likely), determine colonization rates. One caveat, as noted in *Methods*, is that hand weeding that targeted three or four species (depending on the site) was carried out in the plots’ first season, which, by definition, removed some individuals of particular non‐sown species. However, the combination of the facts that the weeding was carried out across all sites, was only conducted in the first year, was targeted at a limited number of key weed species, and was not systematically related to treatment in a way that would produce the observed effects, inclines us to the view that the result is a genuine reflection of treatment effects on colonization and establishment.

The loss of sown species and the establishment of non‐sown species clearly have implications for the longevity of urban meadow plantings and therefore their utility for enhancing urban grassland diversity. Our experiment indicates the potential for change in species composition in the short term, but does not allow longer‐term trajectories to be assessed directly. A number of processes are likely to be important over longer time scales: the disappearance of initially colonizing ruderal species in the absence of further disturbance to the soil (Hofmann and Isselstein 2004, Pywell et al. 2007), the capacity of planted biennial species, e.g., teasel *Dipsacus fullonum,* to self‐seed into the new sward (van der Meijden et al. 1992), the colonization of additional perennial species through dispersal from other sites (Tilman 1997), and the longer term outcomes of competitive interactions between currently established species (Harrison and Bardgett 2010, Maynard et al. 2017). External drivers may also interact with these processes to affect longer‐term outcomes. The plant community will interact with soil microbial and invertebrate communities, and there is emerging evidence that soil microbial communities may facilitate, not simply follow, vegetation development (Harris 2009, van der Putten et al. 2013). Establishing seminatural grasslands in the longer term is, however, also highly dependent on environmental conditions (Stuble et al. 2017), and future management interventions will impact the community trajectory, particularly the frequency of mowing, the choice of whether vegetation clippings are removed (see next paragraph), and any management designed to enhance plant diversity or density, for example re‐seeding or scarifying (Westbury et al. 2006, Trowbridge et al. 2017).

High nitrogen levels of urban soils (Ladd 2016) potentially limit plant diversity. Indeed, two of the five sown plant species (*Primula vulgaris* and *Ranunculus acris*) that failed to become established are very sensitive to high fertility soils, as indicated by their Ellenberg N scores (Hill et al. 1999). The tallest plots consistently had the lowest soil total N, presumably due to greater uptake by growing vegetation, combined with removal of vegetation clippings. Management that includes arising removal should gradually lower soil nitrogen (Walker et al. 2004) and increase the site's suitability for long‐term maintenance of diverse meadow vegetation. However, implementation of such management at larger scales is seen as a major challenge (Hoyle et al. 2017). The tall plots here produced approximately 67 kg (dry mass) per 100 m^2^ of meadow in an annual cut (Appendix S1: Section S4). While there is in principle potential for land managers to use this material in composting or as biomass, this is challenging in urban areas due to concerns regarding contamination with litter and dog feces (Hoyle et al. 2017).

### Invertebrates

Urban meadows typically supported invertebrate communities that were more abundant, diverse, and had greater biomass than those in the unmanipulated amenity grassland. While our measures of invertebrate diversity are based on coarse taxonomic data, higher taxon diversity can indicate trends at finer taxonomic resolutions (Timms et al. 2013, van Rijn et al. 2014), and maintaining a range of orders also has conservation significance. These effects of meadow creation occurred within the meadows’ second growing season suggesting that invertebrate communities could respond rapidly to habitat creation despite potential colonization barriers arising from the fragmented nature of urban greenspace (Braaker et al. 2014, Vergnes et al. 2014).

Effects on invertebrate communities arose more frequently in response to differences in vegetation height rather than plant richness. This is consistent with effects seen in more rural grasslands (Blaauw and Isaacs 2012, Buri et al. 2013, Andrey et al. 2014) and with previous studies of a range of invertebrate groups in which both abundance (Garbuzov et al. 2015) and species diversity (Unterweger et al. 2017) increased with reduced mowing and resultant longer vegetation. We find, however, that taxa varied in their response to medium and tall vegetation with some groups responding more favorably to vegetation of intermediate height in autumn. This could be a response to specific abiotic conditions such as temperature and humidity that vary with sward height and density and will attract and retain species with different requirements than those occurring in longer vegetation (Crist and Ahern 1999, Gardiner and Hassall 2009). Previous work in urban areas focused on shorter swards has found that even relatively small changes in mowing frequency (e.g., every 3 weeks rather than every week (Lerman et al. 2018) can lead to meaningful increases in resources for invertebrates (Shwartz et al. 2013, Lerman et al. 2018). Our work suggests that further increases in vegetation height are likely to yield additional biodiversity benefits. In addition, we note that taller plots were important in winter as well as summer and autumn. It is difficult to tease apart the effects of taller vegetation creating more favorable conditions than short mown grass in winter, from the positive effect of taller vegetation in summer on invertebrate abundance carrying over to influence the winter community. However, the invertebrate abundances in the medium height plots (which were cut low in the winter) were very similar to those of the short plots, and less than the tall plots (uncut; Appendix S1: Fig. S2), suggesting that maintaining longer vegetation during winter is critical. These results emphasize the generally neglected point that consideration of resource availability throughout the year is important for invertebrates (Unterweger et al. 2018), particularly if these areas are to support sustainable populations rather than relying on annual summer recolonization (Leather et al. 1995).

Effects of plant diversity on invertebrate communities were more limited. This may reflect the greater importance of structure, or simply that the structural variation captured a more ecologically significant range of conditions. It may also be a consequence of the taxonomic resolution of the data: at coarse taxonomic resolutions it is harder to detect more specialized responses to particular plant species. The low plant diversity treatment did, however, lack sown forbs and increasing forb cover in higher plant diversity treatments could have contributed to some of the observed responses through the provision of suitable food plants, structural features, and flower‐related resources (nectar, pollen, and seeds). Pollinators, or flower associated species, occur in a number of the Coleoptera families, and other orders that responded to diversity treatments (notably Diptera; Orford et al. 2015). While an equivalent response was not documented in Hymenoptera, which includes a number of pollinators, this order is dominated by parasitic taxa that rarely visit flowers (Shaw and Hochberg 2001). In early autumn, when many flowers were no longer in bloom, the Hemiptera and Thysanoptera were most abundant in the low diversity, grass‐only treatments. This may be due to large numbers of common UK Hemiptera associated with grasses as food plants (Chinery 2012), and that while many thrips are flower dependent, some taxa live in and feed on grass seed heads (Mound and Palmer 1972, Stevenson et al. 1997); indeed, we observed very high abundances of thrips within grass seed heads in our samples.

Declines in invertebrate biomass (Hallmann et al. 2017) and abundance (Conrad et al. 2004, Brooks et al. 2012, Ewald et al. 2015) are of increasing conservation concern, in addition to the loss of species diversity (Fonseca 2009). While it is not possible with these data to tease apart the mechanisms linking changes in plant height and diversity to the changes in the invertebrate community, it is clear that replacing mown amenity grassland with urban meadows can increase the diversity, biomass, and abundance and alter the composition of urban invertebrate communities. It is also clear that invertebrate responses vary according to the particular height and diversity characteristics of the meadows created. This suggests that, at the scale of individual sites or across a network of urban greenspaces, beneficial impacts will be maximized by creating a diverse range of meadow types.

### Soils

The disturbance to the soils during plot establishment, particularly from tilling, combined with the relatively short duration of the experiment compared to the response time for some soil properties, means it is not possible to draw conclusions about long‐term effects of the meadows on the soils. Despite this caveat, soil properties and microbial communities exhibited a number of notable responses to the meadow treatments. The PLFA analysis indicated that, at both soil depths, the composition of soil microbial communities was influenced by plant height and diversity. The DNA results indicated changes to community composition of bacteria and fungi between treatments, although diversity was largely unchanged. The bacterial community composition responded to changes in plant height treatment at both depths. Fungal community composition at 0–10 cm depth responded to plant diversity, and fungi at 11–20 cm depth responded to plant height. The contrasting responses of bacteria and fungi to conditions at different depths, and the closer association of fungal composition with changes in plant diversity, are consistent with previous findings although the driving factors are still poorly understood, ranging from pH, through nitrogen, to antecedent use (Newbound et al. 2012, Xu et al. 2014, Sarah et al. 2015, Yan et al. 2016, Hui et al. 2017). In our study system, recent antecedent use is uniform across treatments suggesting that divergent effects of plant communities on soil nitrogen may contribute to differences in microbial communities. A key change in plant diversity in the treatments was from domination by grasses to domination by forbs, with concomitant changes in the structure and depth of the rooting zone. While bacteria and fungi have complex interactions within the rhizosphere (de Boer et al. 2005), our results suggest a more pronounced shift in the fungal community at shallower depths where the majority of change in the root structure will be observed. Tilling during site preparation may have contributed to the variation in community composition between depths by creating new microhabitats (Bruns 1995), although disturbances similar to tilling can reduce the ability of fungi to establish interactions with host plants (Jasper et al. 1989, McGonigle and Miller 1996). Despite these potential adverse impacts of site management it is clear that replacing mown amenity grassland with meadow style vegetation can alter soil microbial communities, and enhance microbial diversity especially when meadows contain a greater number of plant species.

### Management recommendations for establishing meadows in public greenspaces

We introduced urban meadows in collaboration with local authority partners to investigate the scope for enhancing the attractiveness and quality of sites for people, and enhancing biodiversity and ecological function. Practitioner orientated management guidelines for creating such urban meadows are provided by Hoyle (2016). Our results demonstrate that maintaining meadow communities that are taller and botanically more diverse than short mown grasslands in urban public parks can increase the abundance and richness of invertebrate communities throughout the year, while also altering soil microbial communities. Crucially, meadows with different height and diversity characteristics supported different communities of invertebrates and microbes, and thus a mosaic of meadow types is likely to enhance biodiversity to a greater extent than habitat creation that focuses on replacing mown amenity grassland with just a single type of meadow.

Long‐term maintenance and retention of urban meadows will require acceptance by local residents and willingness on the part of local authorities to maintain them. Previous work on these experimental plots has suggested that creating urban meadows typically increases local residents’ appreciation of the site and perception of site quality, although not all not residents respond favorably (Southon et al. 2017). Local residents generally gave plots with high plant richness and medium height vegetation higher scores for aesthetic preference, compared to short low diversity vegetation that represents mown‐amenity grassland (Southon et al. 2017). Medium height plots do frequently enhance the diversity or abundance of the taxa examined here compared to short treatments, although taller plots were most consistently associated with increased richness and abundance, particularly of invertebrates (Table 3). While the tall treatments were not generally favored by people and were even less attractive to them during winter, Southon et al. (2017) found that people were more prepared to tolerate them when provided with information about their benefits to biodiversity. Consequently, even though there is a perception among some land managers that the public dislike more natural vegetation (Özgüner et al. 2007), our work demonstrates that this is not necessarily the case and there is potential to generate win‐win scenarios for biodiversity and people by introducing biodiverse urban meadows in place of short mown grassland.

Maximizing the potential benefits of urban meadows will require careful consideration of a number of factors. First, our results suggest that maintaining a diversity of meadow types (with varying vegetation height and plant richness) is likely to maximize cumulative biodiversity benefits and the resulting landscape heterogeneity may also further increase the aesthetic appeal of urban meadows (Dramstad et al. 2001). Achieving this is likely to require careful landscape design, yet integrating design principles with the needs of biodiversity is rarely done (Wang et al. 2017). It will also require urban managers to coordinate novel and more complex mowing regimes, which can be challenging, especially when this task is subcontracted to a third party (Hoyle et al. 2017). Second, an important step in introducing taller non‐woody vegetation is communicating intent to the public, for example through on‐site signage. In part, this will increase acceptance among people who are sympathetic to biodiversity conservation, but more generally will help generate the “cues to care” that may be important in increasing public acceptance of wilder vegetation (Nassauer 1995). Creating mown paths that “frame” longer vegetation may further help indicate that meadow areas are under active management and thus cared for. Third, the context of the site was also a factor in public acceptance, with sites directly overlooked by residential housing coming under greater scrutiny (Hoyle et al. 2017) and such sites are thus perhaps less suitable than locations that are less visible from houses or may only be suitable for the most preferred types of urban meadows. Another important aspect of site context is likely to be the proportion of mown amenity grassland converted to urban meadows. Amenity grassland provides important recreational space for exercise and sporting activities, for which urban meadows are not suitable, suggesting it will be best to establish urban meadow vegetation at sites where some short grassland can also be retained. A final challenge in realizing the potential dual benefits of urban meadows for people and biodiversity concerns biomass removal. Reducing mowing and the associated energy and labor costs is one of the assumed benefits of managing green spaces as a perennial meadow rather than as mown grass (Hoyle et al. 2017). At the end of the season, though, all biomass has to be removed, and concerns regarding contamination with litter, dog feces and other material can increase the challenges of using the biomass for energy production of composting although some contractors appear willing to take this material.

We demonstrate that sown perennial meadows can support plant, invertebrate and soil microbial communities both different and more diverse than those of mown amenity grassland. Creating and maintaining urban meadows is not without its challenges but our results suggest that, with careful management and implementation, replacing some of the mown amenity grassland that currently dominates many towns and cities with a range of different types of meadow vegetation can generate positive outcomes for both biodiversity and people.

## Data Availability

Data are available from the Natural Environment Research Council (NERC) at the Environmental Information Data Centre (EIDC): https://doi.org/10.5285/d0741544-cdf3-497d-996b-e30b4b7373c1. DNA data are available as a BioProject from the National Center for Biotechnology Information (NCBI) (accession number PRJNA531648): http://www.ncbi.nlm.nih.gov/bioproject/531648.
