# Supplementary material for: Urban meadows as an alternative to short mown grassland: effects of composition and height on biodiversity
Source: Ecol Appl. 2019 Jul 22;29(6):e01946. doi: 10.1002/eap.1946 (PMC6851864; doi:10.1002/eap.1946)
Supplement: Supplementary file 1 [file EAP-29-na-s001.pdf]

Supporting Information. Norton, Briony A., Gary D. Bending, Rachel Clark, Ron Corstanje, Nigel Dunnett, Karl L. Evans, Darren R. Grafius, Emily Gravestock, Samuel M. Grice, Jim A. Harris, Sally Hilton, Helen Hoyle, Edward Lim, Theresa G. Mercer, Mark Pawlett, Oliver L. Pescott, J. Paul Richards, Georgina E. Southon, and Philip H. Warren. 2019. Urban meadows as an alternative to short mown grassland: effects of composition and height on biodiversity. *Ecological Applications.*

Appendix S1

Figure S1: Maps showing the location of the experimental sites and their surrounding landscape. (A – Luton site; B – Bedford sites; C – Cranfield University site).

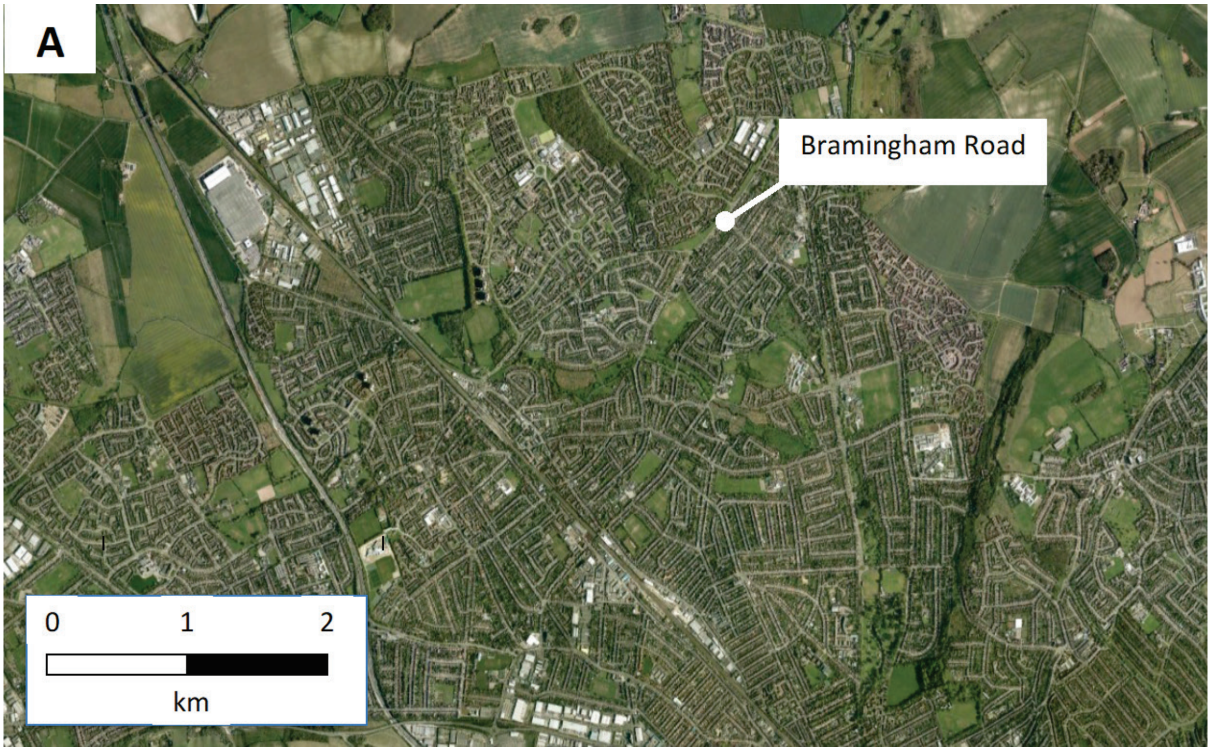

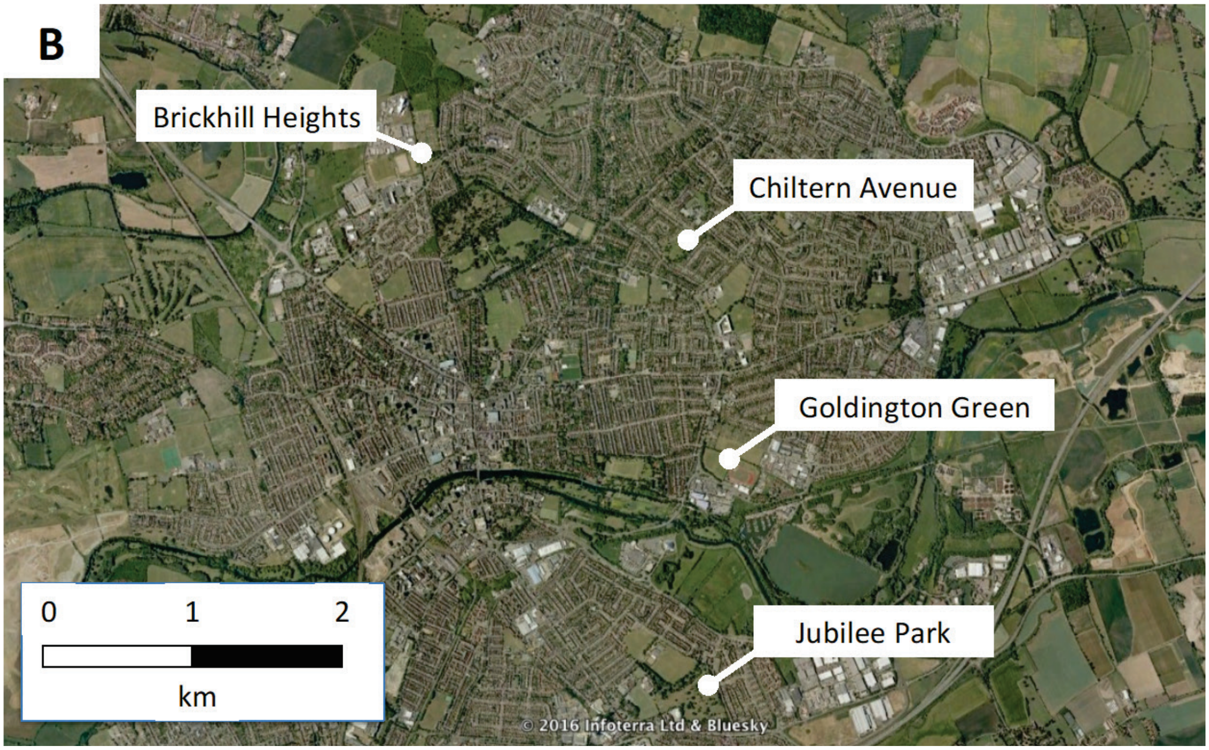

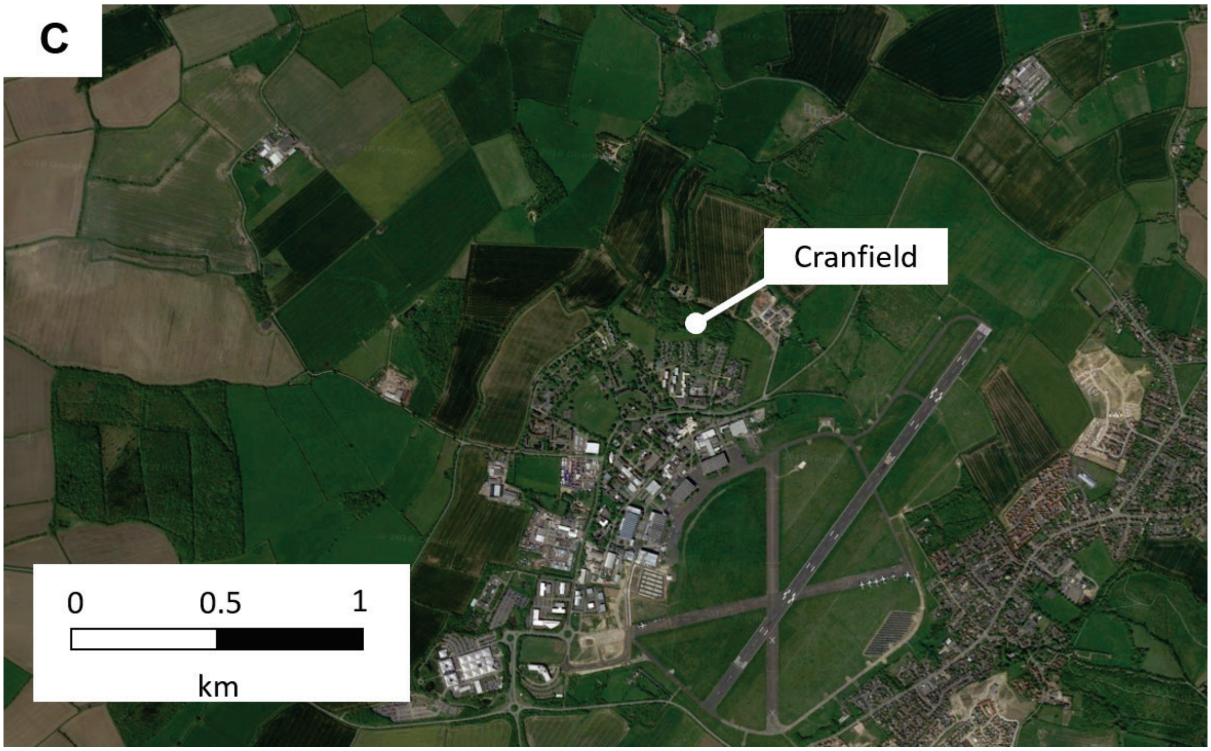
C
Cranfield
0
0.5
1
km

Table S1: Site details and invertebrate sampling dates in treatment year 2. For details of treatments, refer to Figure 1 in the main manuscript.

Site details					Invertebrate sampling dates treatment year 2		
**Town**	**Site**	**Location**	**Treatment set**	**Plot size**	**Summer**	**Autumn**	**Winter**
Bedford	Chiltern Ave	52°08'53.2"N 0°26'48.0"W	Full	12.5 m x 20 m	17 June 2014	2 September 2014	11th and 13th February 2015
Bedford	Goldington Green	52°08'38.9"N 0°25'42.0"W	Excludes: H1.D2 H2.D2 H3.D2	12.5 m x 20 m	18 June 2014	3 September 2014	9 February 2015 (only unmanipulated control + H3.D1 + H3.D3 available, others rotovated)
Bedford	Jubilee Park	52°07'18.0"N 0°26'50.3"W	Full	12.5 m x 20 m	23 July 2015	7 September 2015	NA
Bedford	Brickhill Heights	52°09'10.2"N 0°28'15.3"W	Excludes H1.D2 H2.D1 H2.D2 H2.D3	12.5 m x 20 m	18 June 2014	2 September 2014	10 February 2015
Luton	Bramingham Road	51°54'53.6"N 0°26'16.2"W	Full	12.5 m x 20 m	17 June 2014	3 September 2014	12 February 2015
Cranfield	Cranfield University	52°04'40.1"N 0°37'36.0"W	Full (small plots)	5 m x 10 m	NA	NA	NA

Table S2: Composition of seed mix per treatment. Treatments are designated by H.D. H = height and D = diversity. H: 1 = short; 2 = intermediate; 3 = tall. D: 1 = low; 2 = medium; 3 = high. Treatments D2 and D3 (grass/forb mixes) plots were initially sown at 4g/m^2, and bare patches were over-sown at the end (autumn) of the first growing season at the same density. Treatment D1 (grass only) plots were initially sown at 4g/m^2 and most (two plots were excluded as they established adequately: H3 at Goldington Green and Bramingham) were sprayed and re-sown at 20 g/m^{2}at the end (autumn) of the first growing season.

**Treatment**	**H1.D1**	**H1.D2**	**H1.D3**	**H2.D1**	**H2.D2**	**H2.D3**	**H3.D1**	**H3.D2**	**H3.D3**
Total Richness	4	9	16	3	10	17	5	13	21
Forb Richness	0	5	12	0	7	14	0	8	16
Grass Richness	4	4	4	3	3	3	5	5	5
Forb: grass seed ratio	All grass	1:3	1:1	All grass	1:3	1:1	All grass	1:3	1:1
**Forb species**	**% of forb community per species (by seed weight)**								
Achillea millefolium		10	5		5	1		10	4
Anthriscus sylvestris								10	4
Arctium minus								4	2
Centaurea nigra					30	10		40	15
Centaurea scabiosa						15			
Daucus carota						3			
Dipsacus fullonum								6	4
Echium vulgare									10
Galium album (mollugo)									7
Galium verum			5		5	2			
Geranium pratense						7			
Hypericum perforatum						1			
Knautia arvensis					25	15			10
Leontodon hispidus			10		15	10			
Leucanthemum vulgare			5			5		7	3
Linaria vulgaris									3
Lotus corniculatus		40	20						
Malva moschata						20			7
Medicago lupulina		20	12						
Ononis spinosa									10
Plantago lanceolata								8	3
Plantago media			3		5	2			
Primula veris			5						
Prunella vulgaris		15	7						
Ranunculus acris			15						
Rumex acetosa		15	8					15	5
Tanacetum vulgare									5
Trifolium pratense			5		15	4			

Vicia cracca						5			8
**Grass species**	**% of grass community per species (by seed weight)**								
Agrostis capillaris				10	10	10			
Agrostis castellana	10	10	10	10	10	10			
Dactylis glomerata							30	30	30
Festuca arundinacea							15	15	15
Festuca pratensis							25	25	25
Festuca rubra	50	50	50	80	80	80	20	20	20
Lolium perenne	20	20	20						
Phleum pratense							10	10	10
Poa pratensis	20	20	20						

Table S3: Invertebrate biomass calculations

Biomass estimates for invertebrates sampled in the meadow experiment were obtained using data from invertebrate samples taken across Bedford, Luton and Milton Keynes in the summer (July and August) of 2013. Invertebrates were sampled in 243 plots (each 25 m^2 in area) across 78 urban green spaces using a sweep net and vacuum sampler, identified to order level primarily (see table for details) and stored in separate vials for each taxon. Of this collection, samples from 18 sites that reflected a range of vegetation types and site sizes were selected to estimate biomass. From this subsample, up to 48 individuals (based on availability of specimens) were selected from each taxon, excepting Acari, which had not been separated from the samples. Length of each individual was measured under the microscope with a graticule to the nearest 1/10th mm. Specimens were then oven-dried overnight (16 hours) at 60° C. Each individual organism was weighed on a microbalance (in mg to 4 dp). Collembola were too light to measure individually, and were instead weighed in groups of 24 and an average weight derived. Where there were too few specimens (< 5) to derive an average weight for a particular taxon, an average weight was derived as a surrogate, using the weights of taxa with similar physical properties.

Dry weight per individual organism in a taxon used to estimate biomass across the treatment plots. The number of specimens is the number used to derive the dry weight. Where this is blank, a surrogate was used, indicated in the final column.

Phylum	Class	Order	lower taxon.1	lower taxon.2	Dry weight (mg)	No. specimens	Weight surrogate
Mollusca	Gastropoda				0.746		Average of all 'large' (> 0.1 mg) taxa
Arthropoda	Arachnida	Acari			0.054		Average of all 'small' (< 0.1 mg) taxa
Arthropoda	Arachnida	Araneae			0.815	36	
Arthropoda	Arachnida	Opiliones			0.815		Araneae
Arthropoda	Arachnida	Pseudoscorpiones			0.746		Average of all 'large' (> 0.1 mg) taxa
Arthropoda	Malacostraca	Isopoda			0.746		Average of all 'large' (> 0.1 mg) taxa
Arthropoda	Chilopoda				0.746		Average of all 'large' (> 0.1 mg) taxa
Arthropoda	Diplopoda				0.746		Average of all 'large' (> 0.1 mg) taxa
Arthropoda	Symphyla				0.009		Thrips
Arthropoda		Collembola			0.061	138	
Arthropoda		Diplura			0.009		Thrips

Arthropoda	Insecta	Coleoptera	adult		1.015	71	
Arthropoda	Insecta	Coleoptera	larvae		0.082	22	
Arthropoda	Insecta	Dermaptera			2.329	8	
Arthropoda	Insecta	Diptera	adult		0.227	56	
Arthropoda	Insecta	Diptera	larvae		0.404	24	
Arthropoda	Insecta	Ephemeroptera			0.515		Average of all adult insects
Arthropoda	Insecta	Hemiptera	Heteroptera		1.128	35	
Arthropoda	Insecta	Hemiptera	Auchenorrhyncha	Hoppers	1.075	35	
Arthropoda	Insecta	Hemiptera	Sternorrhyncha	Aphids	0.064	36	
Arthropoda	Insecta	Hemiptera	Sternorrhyncha	Other	0.064		Aphids
Arthropoda	Insecta	Hymenoptera	Formicidae		0.313	24	
Arthropoda	Insecta	Hymenoptera	Other		0.059	48	
Arthropoda	Insecta	Lepidoptera	adult		1.026	3	
Arthropoda	Insecta	Lepidoptera	larvae		0.137	16	
Arthropoda	Insecta	Mecoptera			0.515		Average of all adult insects
Arthropoda	Insecta	Neuroptera	adult		0.515		Average of all adult insects
Arthropoda	Insecta	Neuroptera	larvae		0.221		Average of all larval insects
Arthropoda	Insecta	Odonata			0.515		Average of all adult insects
Arthropoda	Insecta	Orthoptera			0.515		Average of all adult insects
Arthropoda	Insecta	Plecoptera	larvae		0.221		Average of all larval insects
Arthropoda	Insecta	Psocodea			0.057	21	
Arthropoda	Insecta	Thysanoptera			0.009	47	
Arthropoda	Insecta	Trichoptera			0.515		Average of all adult insects
Arthropoda	Insecta	Zygentoma			0.009		Thrips

Section S1: Extended method details: supplement to main manuscript Methods: Invertebrates

Aboveground invertebrates were sampled twice in all plots: first in the summer (June/July) and then the autumn (early September) of the second year post-sowing. Sampling was scheduled to occur at least a week after mowing, but unplanned changes in the council maintenance schedule meant that a subset of plots (n = 4, September) had been recently mown prior to sampling. Sweep netting (primarily targeting taxa that can fly) was undertaken along a 20 m transect through the middle of the plot, using a white net (60 cm diameter). Vacuum sampling (for invertebrates on the ground and vegetation) was undertaken at two points in each plot using a cordless leaf blower/vac (airspeed = 320 km/h; 11 cm diameter nozzle). Sampling locations were away from the sweep net transect and at least 2 m apart. Sampling was conducted at all vegetation heights in a sampling container (0.5 m in diameter and 1.5 m high) that prevented the escape of aerial insects.

The potential of meadows to support overwintering invertebrates was assessed through sampling conducted in February 2016 at sites at the end of their second-year post-establishment (Brickhill Heights, Chiltern Avenue, Bramingham Road and Goldington Green). At Goldington Green only three plots were available for sampling as four plots had experienced unscheduled rotovation by greenspace management teams. Sweep netting was conducted as in summer but vegetation was too compacted and damp for effective use of the blower/vac. Hand-searching was thus conducted in two, 1 m^2 quadrats in each plot, off the sweep net transect and at least 2 m apart. Searches involved a sequence of beating, cutting, collecting and sieving vegetation. A pooter/aspirator was used to extract invertebrates from the ground surface and collected vegetation, by orally sucking them into collecting tubes. Each quadrat was surveyed for a maximum of 50 minutes, with equal time allocated to each quarter of the quadrat and a stopping rule used for each survey method.

All invertebrate specimens were stored in 80% industrial methylated spirits prior to identification to order (class for Chilopoda, Diplopoda and Gastropoda) using a binocular microscope. Order is a coarse resolution, but sorting to this level allowed an assessment of all collected taxa simultaneously at a level directly comparable between sites and studies. The numbers of each taxon were counted precisely except where one taxonomic group (usually Acari or Collembola) exceeded approximately 200 individuals, when they were estimated by precisely counting two or three groups of 10 or 100 individuals (depending on abundance) and then estimating the number of such groups in the entire sample. Adults and larvae from each taxon were combined to order level for analysis. Coleoptera are a taxonomically and functionally diverse order of insects, and were ubiquitous across plots. Coleoptera adults from the summer and autumn samples and from the three sites with a full set of treatments (Chiltern Avenue, Bramingham Road, Jubilee Park) were identified to family to provide a taxonomically and functionally more finely resolved assessment of differences in diversity and composition across the plots. Finally, invertebrate biomass was estimated based on an average oven-dry weight of approximately 30 individuals for each order, or the closest taxonomic grouping if too few specimens were available, using samples collected from greenspace in the surrounding urban landscape (Table S3).

Section S2: Extended method details: supplement to main manuscript Methods: Soils, Soil biological community

Microbial biomass-C was determined using the fumigation-extraction procedure (Jenkinson and Powlson 1976) using the KEC of 0.45 (Vance et al. 1987). Microbial community phenotypic characteristics were determined by analysing cellular phospholipids using an adaptation of Frostegård's et al. (1993) method. Phospholipid Fatty Acids (PLFA) were extracted from approximately 7 g freeze dried soil. Fatty acids were derivatised by mild alkaline methanolysis prior to analysis by gas chromatography (Agilents, USA) using a HP-5 (Agilent Technologies) capillary column (30 m length, 0.32 mm ID, 0.25 µm film). The GC conditions were reported in Pawlett et al. (2013). Fatty acids were identified by comparison of sample retention time to a standard qualitative bacterial acid methyl ester mix (Supelco) and by using gas chromatography coupled with mass spectroscopy (Agilents, USA). Resultant fatty acids were integrated using G2070 ChemStation for G.C, and calculated as relative abundance (mol %). The mol% of indicator fatty acids was used as an indicator of the presence of group of organisms. Indicator fatty acids included: 18:2ω6, 9 (ectomycorrhizal fungi) and the sum of i15:0, ai15:0, 15:0, 16:1, i16:0, 16:1ω9, 16:1ω7 t, i17:0, ai17:0, cyc-17:0, 17:0 and cyc-19:0 (total bacteria).

Total DNA was extracted from 250 mg soil using the PowerSoil DNA Isolation kit (MoBio, USA) according to the manufacturer's instructions. For each sample, 10 ng of DNA was used to amplify either the ITS region using the ITS fungal primer pair ITS3 and ITS4 (White et al. 1990), or the 16S rRNA gene using the 16S bacterial primer pair 515f and 806r (Caporaso et al. 2011). The two primer sets were modified at the 5′ end with Illumina Nextera Index Kit v2 adapters. PCR reactions were performed in a reaction volume of 25 µl, containing Q5® Hot Start High-Fidelity 2X Master Mix (New England Biolabs) and 0.5 µM of each primer. Thermocycling consisted of an initial denaturation at 98°C for 30 s followed by 30 cycles of 98°C for 10 s, 50/57°C (16S/ITS) for 15 s and 72°C for 20 s with a final extension at 72°C for 5 min. The libraries were sequenced using the Illumina MiSeq Reagent Kit v3 (600-cycle). Following sequencing, Trimmomatic v0.35 was used to remove low-quality bases from the sequence ends (Bolger et al. 2014). The following steps were then performed using USEARCH and UPARSE software (Edgar 2010, 2013). Paired-ends reads were assembled by aligning the forward and reverse reads, trimming primers and quality filtering (-fastq_maxee 0.5). Unique sequences were sorted by abundance and singletons were discarded from the dataset. Sequences were clustered to Operational Taxonomic Units (OTUs) at 97% minimum identity threshold. Taxonomy was assigned using Quantitative Insights into Microbial Ecology (QIIME 1.8) (Caporaso et al. 2010) and the Greengenes reference database for 16S (McDonald et al. 2011), or the UNITE database for ITS (Kõljalg et al. 2013).

Table S4: Total abundance of each aboveground invertebrate taxon collected in summer, autumn and winter in the second growing season of the meadow treatments. Collection methods in winter were different, from those in summer and autumn (see main manuscript Methods: Invertebrates and Appendix S1: Sect. S1) and sampling was undertaken on a subset of sites (Table S1).

Taxon	Summer	Autumn	Winter
Chilopoda	4	16	2
Diplopoda	7	21	8
Gastropoda	138	533	455
Acari	5298	19204	173
Araneae	1523	3612	397
Coleoptera	8095	2953	253
Collembola	19424	25727	1,724
Dermaptera	91	53	2
Diplura	2	5	0
Diptera	8055	5607	705
Ephemeroptera	0	2	0
Hemiptera	10283	9970	283
Hymenoptera	2803	3421	28
Isopoda	38	34	0
Lepidoptera	112	185	74
Neuroptera	32	14	0
Odonata	31	1	0
Opiliones	0	5	0
Orthoptera	1	2	0
Psocodea	172	2322	154
Thysanoptera	3232	5277	149
Zygentoma	0	1	0

Table S5: Output from adonis and betadisper models (vegan, Oksanen et al. 2017) for plants and invertebrates (summer and autumn order-level community and Coleoptera families). Model terms are added sequentially and treatment effects are reported for the model where the other treatment is accounted for (for details refer to main manuscript Methods: Data Analysis, Plants and invertebrates – ordinations). Site was included as a blocking factor. The betadisper p-values indicate whether there is significant differences in homogeneity of group variances. Where these are < 0.05, the adonis outputs should be treated cautiously as they as they are sensitive to variation in homogeneity of group variances.

adonis							betadisper
**Taxon**		**Df**	**SumsOfSqs**	**F.Model**	**R2**	**Pr(>F)**	**Pr(>F)**
Plants All	Site: Diversity	9	3.5744	1.7705	0.27741	**0.001**	0.173
	Site: Height	9	4.0785	2.0202	0.31653	**0.001**	0.362
	Site	4	1.8038	2.0103	0.13999	**0.001**	
	Residuals	15	3.3649		0.26114		
	Total	37	12.8851		1		
Plants Non-sown	Site: Diversity	9	3.3999	1.2431	0.22999	**0.045**	0.709
	Site: Height	9	3.6194	1.3234	0.24484	**0.018**	**0.010**
	Site	4	3.1108	2.5592	0.21043	**0.01**	
	Residuals	15	4.5583		0.30835		
	Total	37	14.783		1		
Order-level community Summer	Site: Diversity	9	0.9272	1.3903	0.20385	0.094	0.313
	Site: Height	9	1.1345	1.7012	0.24943	**0.026**	0.104
	Site	4	1.3421	4.5281	0.29507	**0.018**	
	Residuals	15	1.1115		0.24437		
	Total	37	4.5484		1		
Order-level community Autumn	Site: Diversity	9	1.0181	1.5411	0.22545	0.060	0.608
	Site: Height	9	1.1641	1.7622	0.25778	**0.023**	0.609
	Site	4	1.1953	4.0713	0.2647	**0.007**	
	Residuals	15	1.101		0.24381		
	Total	37	4.5159		1		
Coleoptera Families Summer	Site: Diversity	6	1.8413	1.8719	0.28478	0.067	**0.010**
	Site: Height	6	1.6155	1.6424	0.24986	0.102	0.727
	Site	2	1.0415	3.1765	0.16108	0.051	
	Residuals	12	1.9673		0.30427		
	Total	26	6.4657		1		
Coleoptera Families Autumn	Site: Diversity	6	1.1714	1.5197	0.24517	0.073	0.776
	Site: Height	6	1.5559	2.0186	0.32566	**0.007**	0.278
	Site	2	0.5089	1.9805	0.1065	**0.008**	
	Residuals	12	1.5416		0.32266		
	Total	26	4.7778		1		

Figure S2: Boxplot of total abundance per plot by height treatment (H1 = short, H2 = medium, H3 = tall) of invertebrates collected during winter surveys (February 2015).

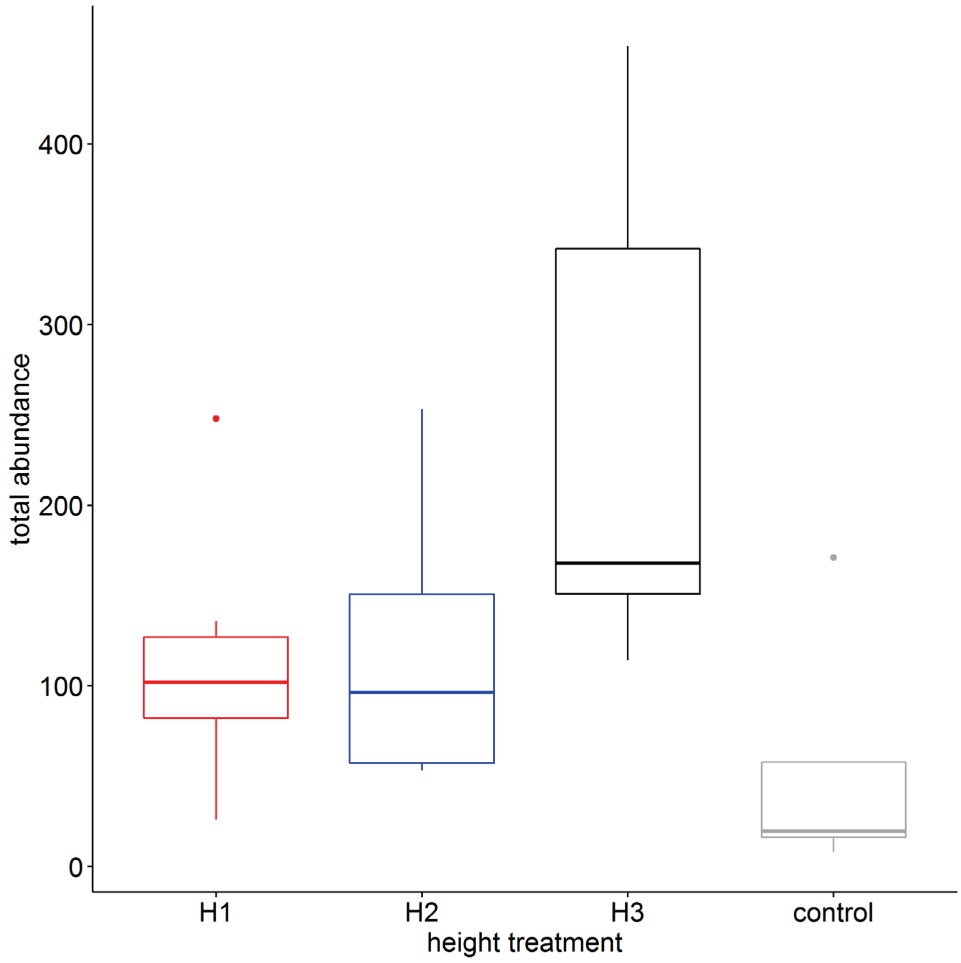

Figure S3: (a) Total nitrogen, and (b) total carbon (both measured as percent dry weight), by treatment and by depth. Bars are organised from short (left) to tall (right) treatments, with small gaps between the height groups, and diversity treatment is indicated by grey shading; light grey = low diversity, medium grey = medium, black = high diversity. White bars represent the unmanipulated control. Bars are the mean per treatment combination with standard deviation bars.

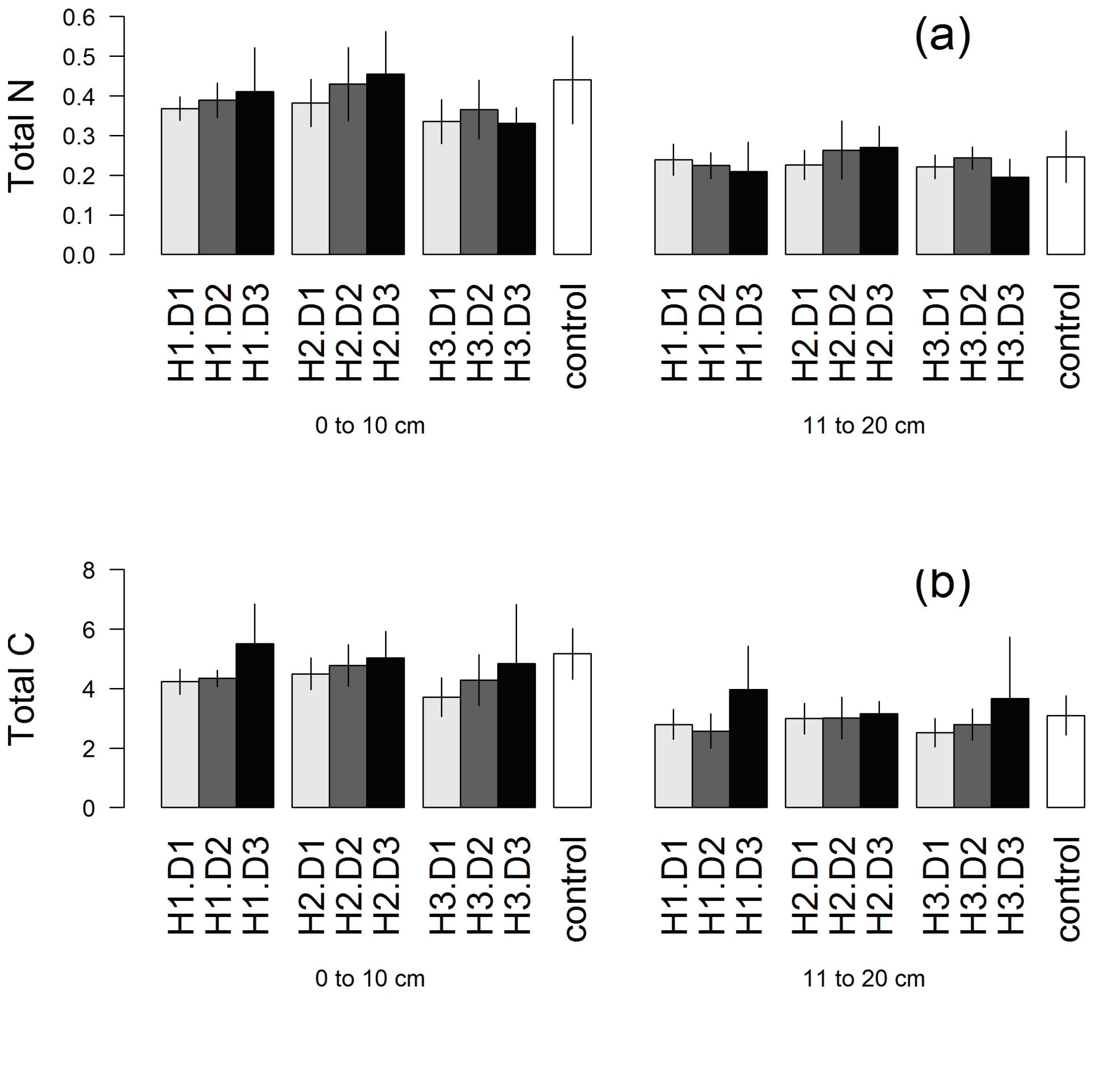

Table S6: Non-sown plant species detected in treatment year 2 and their lifecycle and strategy according to Grime's CSR theory (Grime 1979, Grime et al. 1995). Each species is denoted as C = competitor, S = stress tolerator, R = ruderal, or some combination of these. ND indicates no data are available. Life cycles are denoted as per = perennial, ann = annual, biann = biannual.

Species	Strategy	Lifecycle
Achillea millefolium	CR	per
Agrostis capillaris	CR	per
Agrostis castellana	ND	per
Agrostis gigantea	CR	per
Agrostis stolonifera	CR	per
Alopecurus myosuroides	ND	ann
Anagallis arvensis	R	ann
Anisantha sterilis	R	ann
Arrhenatherum elatius	C	per
Ballota nigra	ND	per
Bellis perennis	R	per
Capsella bursa pastoris	R	ann
Centaurea nigra	CSR	per
Centaurea scabiosa	S	per
Cerastium fontanum	R	ann
Chenopodium album	R	ann
Chenopodium polyspermum	ND	ann
Cirsium arvense	C	per
Cirsium vulgare	CR	biann
Convolvulus arvensis	CR	per
Conyza canadensis	ND	ann
Crepis capillaris	R	ann
Cynosurus cristatus	CSR	per
Dactylis glomerata	C	per
Daucus carota	ND	biann
Echium vulgare	ND	biann
Elytrigia repens	C	per
Epilobium sp.	ND	ND
Epilobium ciliatum x cf. *tetragonum*	ND	per
Epilobium obscurum	CSR	per
Epilobium tetragonum	ND	per
Fallopia convolvulus	ND	ann
Festuca arundinacea	CSR	per
Festuca pratensis	CSR	per
X *Festulolium loliaceum*	ND	per
Galium aparine	CR	ann

Galium mollugo	ND	ann
**Species**	**Strategy**	**Lifecycle**
Galium sp.	ND	ND
Galium verum	CS	per
Geranium dissectum	R	ann
Geranium molle	R	ann
Geranium pratense	ND	per
Geum urbanum	S	per
Helictotrichum pratense	S	per
Holcus lanatus	CSR	per
Hordeum murinum	ND	ann
Knautia arvensis	CSR	per
Lactuca serriola	R	ann
Lactuca virosa	R	ann
Lamium album	CR	per
Lapsana communis	R	ann
Leucanthemum vulgare	C	per
Lolium perenne	CR	per
Lotus corniculatus	S	per
Malva moschata	CSR	per
Medicago lupulina	R	per
Myosotis arvensis	R	ann
Papaver rhoeas	R	ann
Phleum pratense	ND	per
Picris echioides	ND	ann
Plantago lanceolata	CSR	per
Plantago major	R	per
Poa annua	R	ann
Poa pratensis	CSR	per
Poa trivialis	CR	per
Polygonum aviculare	R	ann
Potentilla reptans	CR	per
Prunella vulgaris	CSR	per
Quercus cerris	ND	per
Ranunculus repens	CR	per
Rumex acetosa	CSR	per
Rumex acetosella	SR	per
Rumex crispus	R	per
Rumex obtusifolius	CR	per
Senecio vulgaris	R	ann
Sherardia arvensis	R	ann

Silene alba	ND	ann
Silene dioica	CSR	per
Silene latifolia ssp. *alba*	R	ann
**Species**	**Strategy**	**Lifecycle**
Sisymbrium officinale	R	ann
Sonchus arvensis	CR	per
Sonchus asper	R	ann
Sonchus oleraceus	R	ann
Stellaria media	R	ann
Taraxacum agg.	R	per
Torilis nodosa	ND	ann
Trifolium dubium	R	ann
Trifolium pratense	CSR	per
Trifolium repens	CR	per
Tussilago farfara	CR	per
Urtica dioica	C	per
Veronica arvensis	R	ann
Veronica chamaedrys	CSR	per
Veronica filiformis	R	per
Veronica persica	R	ann
Vicia cracca	C	per
Viola arvensis	R	ann
Viola tricolor	R	ann
Vulpia bromoides	SR	ann

Section S3: Bare ground cover extended results details: supplement to main manuscript Discussion: Plants

Figure S4: Percent of bare ground cover (mean and standard deviation) in the experimental treatments and unmanipulated control. Bare ground cover was recorded as part of the plant surveys, using Domin cover estimates (for details see main manuscript Methods: Botanical surveys).

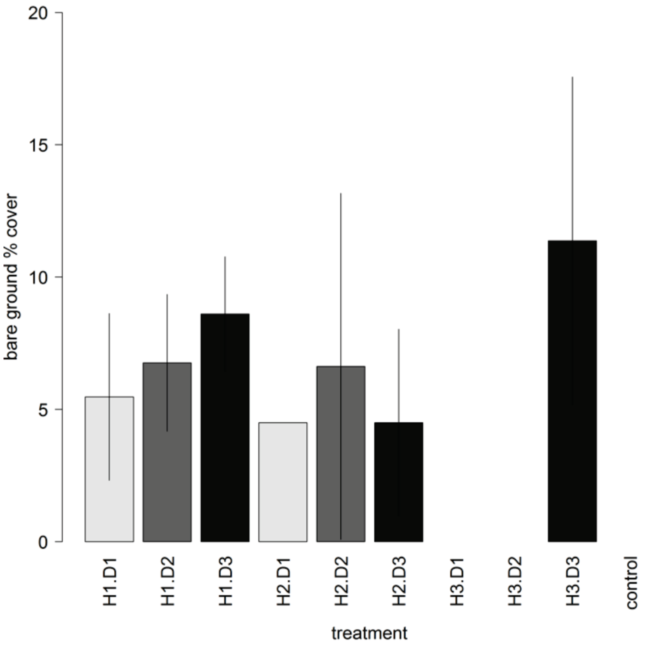

Linear mixed effects models were constructed in lme4 (Bates et al. 2015) with height (three levels) and diversity (three levels) treatments included as fixed effects and site as a random intercept, using maximum likelihood parameter estimation. P-values for the main fixed effects were extracted using the package lmerTest (Kuznetsova et al. 2017), with degrees of freedom based on Satterthwaite's approximation. Within-treatment pairwise comparisons were determined with post-hoc tests using least-squares means in package lsmeans (Lenth 2016).

No significant effect of treatment on bare ground cover was detected (Diversity: $F_{2,19.0}$ = 0.91, P = 0.421; Height: $F_{2,19.0}$ = 2.10, P = 0.150).

Section S4: Vegetation clippings biomass extended results details: supplement to main manuscript Discussion: Plants

Method

We estimated biomass of vegetation clippings (arisings) from a complete cut of the medium and tall plots prior to termination of the experiment (3 November 2015). Samples were taken from Chiltern Avenue (year 3 post-sowing) and Jubilee Park (year 2 post-sowing). These sites were selected as they retained a full set of un-mown treatments at this point of sampling.

In each medium and tall treatment plot at each site, all vegetation within two 0.5 m x 1 m quadrats was cut off at the base and collected in to large, plastic bags. Quadrats were placed to capture remaining areas of non-trampled vegetation. Samples were stored in freezers for two days prior to processing.

All samples were weighed wet, cut into pieces and thoroughly mixed up. A subsample of each was oven-dried at 60°C for 60 hours. Subsamples were weighed before and after drying to measure moisture content of the sample. The moisture loss from the subsample combined with the final dry weight was used to back-calculate the total estimated dry weight of each sample. The two plot replicates were combined to estimate dry weight per 1 m^2 per plot.

Results

Table S7: Estimated dry weight in kilograms per 1 m^2 of vegetation clippings from medium (H2) and tall (H3) experimental meadow plots. D1 to D3 represent the low to high diversity treatments (Figure 1).

Site	H2.D1	H2.D2	H2.D3	mean H2	H3.D1	H3.D2	H3.D3	mean H3
**Chiltern Ave**	0.29	0.26	0.22	**0.26**	0.66	0.71	0.80	**0.72**
**Jubilee Park**	0.55	0.26	0.52	**0.44**	0.61	0.66	0.59	**0.62**
**Mean (kg)**				**0.35**				**0.67**

References: Appendices

Bates, D., M. Maechler, B. Bolker, and S. Walker. 2015. Fitting Linear Mixed-Effects Models Using lme4. Journal of Statistical Software **67**:1-48.

Bolger, A. M., M. Lohse, and B. Usadel. 2014. Trimmomatic: a flexible trimmer for Illumina sequence data. Bioinformatics **30**:2114-2120.

Caporaso, J. G., J. Kuczynski, J. Stombaugh, K. Bittinger, F. D. Bushman, E. K. Costello, N. Fierer, A. G. Peña, J. K. Goodrich, J. I. Gordon, G. A. Huttley, S. T. Kelley, D. Knights, J. E. Koenig, R. E. Ley, C. A. Lozupone, D. McDonald, B. D. Muegge, M. Pirrung, J. Reeder, J. R. Sevinsky, P. J. Turnbaugh, W. A. Walters, J. Widmann, T. Yatsunenko, J. Zaneveld, and R. Knight. 2010. QIIME allows analysis of high-throughput community sequencing data. Nature Methods **7**:335–336.

Caporaso, J. G., C. L. Lauber, W. A. Walters, D. Berg-Lyons, C. A. Lozupone, P. J. Turnbaugh, N. Fierer, and R. Knight. 2011. Global patterns of 16S rRNA diversity at a depth of millions of sequences per sample. Proceedings of the National Academy of Sciences **108**:4516-4522.

Edgar, R. C. 2010. Search and clustering orders of magnitude faster than BLAST. Bioinformatics **26**:2460-2461.

Edgar, R. C. 2013. UPARSE: highly accurate OTU sequences from microbial amplicon reads. Nature Methods **10**:996.

Frostegård, Å., A. Tunlid, and E. Bååth. 1993. Phospholipid fatty acid composition, biomass, and activity of microbial communities from two soil types experimentally exposed to different heavy metals. Applied and Environmental Microbiology **59**:3605-3617.

Grime, J. P. 1979. Plant Strategies and Vegetation Processes. Wiley and Sons, Chichester, UK.

Grime, J. P., J. G. Hodgson, R. Hunt, and K. Thompson. 1995. The Electronic Comparative Plant Ecology. Chapman & Hall, London, UK.

Jenkinson, D. S., and D. S. Powlson. 1976. The effects of biocidal treatments on metabolism in soil—I. Fumigation with chloroform. Soil Biology and Biochemistry **8**:167-177.

Kõljalg, U., R. H. Nilsson, K. Abarenkov, L. Tedersoo, A. F. S. Taylor, M. Bahram, S. T. Bates, T. D. Bruns, J. Bengtsson-Palme, T. M. Callaghan, B. Douglas, T. Drenkhan, U. Eberhardt, M. Dueñas, T. Grebenc, G. W. Griffith, M. Hartmann, P. M. Kirk, P. Kohout, E. Larsson, B. D. Lindahl, R. Lücking, M. P. Martín, P. B. Matheny, N. H. Nguyen, T. Niskanen, J. Oja, K. G. Peay, U. Peintner, M. Peterson, K. Põldmaa, L. Saag, I. Saar, A. Schüßler, J. A. Scott, C. Senés, M. E. Smith, A. Suija, D. L. Taylor, M. T. Telleria, M. Weiss, and K. H. Larsson. 2013. Towards a unified paradigm for sequence-based identification of fungi. Molecular Ecology **22**:5271-5277.

Kuznetsova, A., P. B. Brockhoff, and R. H. B. Christensen. 2017. lmerTest Package: Tests in Linear Mixed Effects Models. Journal of Statistical Software **82**:1-26.

Lenth, R. V. 2016. Least-Squares Means: The R Package lsmeans. Journal of Statistical Software **69**:1-33.

McDonald, D., M. N. Price, J. Goodrich, E. P. Nawrocki, T. Z. DeSantis, A. Probst, G. L. Andersen, R. Knight, and P. Hugenholtz. 2011. An improved Greengenes taxonomy with explicit ranks for ecological and evolutionary analyses of bacteria and archaea. The Isme Journal **6**:610.

Oksanen, J., F. G. Blanchet, M. Friendly, R. Kindt, P. Legendre, D. McGlinn, P. R. Minchin, R. B. O'Hara, G. L. Simpson, P. Solymos, M. H. H. Stevens, E. Szoecs, and H. Wagner. 2017. vegan: Community Ecology Package. R package version 2.4-2.

Pawlett, M., K. Ritz, R. A. Dorey, S. Rocks, J. Ramsden,and J. A. Harris. 2013. The impact of zero-valent iron nanoparticles upon soil microbial communities is context dependent. Environmental Science and Pollution Research **20**:1041-1049.

Vance, E. D., P. C. Brookes, and D. S. Jenkinson. 1987. An extraction method for measuring soil microbial biomass C. Soil Biology and Biochemistry **19**:703-707.

White, T. J., T. Bruns, S. Lee, and J. Taylor. 1990. Amplification and direct sequencing of fungal ribosomal RNA genes for phylogenetics. Pages 315-322 in M. A. Innis, D. H. Gelfand, J. J. Sninsky, and T. J. White, editors. PCR protocols: a guide to methods and applications. Academic Press Inc., New York.
